# Synthesis, Biological Evaluation, and QSAR Studies of 3-Iodochromone Derivatives as Potential Fungicides

**DOI:** 10.3389/fchem.2021.636882

**Published:** 2021-04-30

**Authors:** Parshant Kaushik, Najam A. Shakil, Virendra S. Rana

**Affiliations:** Division of Agricultural Chemicals, ICAR-Indian Agricultural Research Institute, New Delhi, India

**Keywords:** 3-iodochromone, fungicidal activity, sclerotium rolfsii, QSAR, multiple linear regression, principle component analysis, partial least square

## Abstract

Despite the emergence of novel biotechnological and biological solutions, agrochemicals continue to play an important role in crop protection. Fungicide resistance is becoming a major problem; numerous cases of fungicide resistance have occurred worldwide in the last decade, resulting in the loss of several fungicides. The discovery of new molecules has therefore assumed critical importance in crop protection. In our quest for biologically active molecules, we herein report the synthesis of a series of twenty-one 3-Iodochromone derivatives (4a–4u), in a two-step process by condensation of 2-hydroxyacetophenone derivatives (2a–2u) with *N*,*N*-dimethylformamidedimethylacetal yielding enaminones (3a–3u), followed by cyclization with iodine to corresponding 3-iodochromones. Characterization of these compounds was done by IR, ^1^H NMR, ^13^C NMR, and LC-HRMS techniques. All synthesized compounds were screened for their fungicidal activity against *Sclerotium rolfsii*. Among these 6,8-Dichloro-3-iodochromone **4r** was found to be most active (ED_50_ = 8.43 mg L^−1^). 2D-Quantitative Structural Activity Relationship (2D-QSAR) analysis was also performed by generating three different models *viz*., Multiple Linear Regression (MLR, Model 1), Principal Component Regression (PCR, Model 2), and Partial Least Squares (PLS, Model 3). Predictive power and statistical significance of these models were assessed with external and internal validation and leave one-out cross-validation was used for verification. In QSAR study, MLR (Model 1) was found to be best having correlation coefficient (r^2^) 0.943, cross-validated correlation coefficient (q^2^) 0.911 and r^2^pred 0.837. It was observed that DeltaEpsilonC, T_2_Cl_6, T_2_F_6, T_T_F_3, and ZCompDipole are the major descriptors which influence the fungicidal activity of 3-Iodochromone derivatives. The physicochemical parameters were estimated by the VLifeMDS 4.6 software. The QSAR study results will be helpful for structure optimization to improve the activity.

## Introduction

The growth of human civilization has been closely related to crop production, and plant diseases have been a concern for human being perhaps since plants were cultivated more than 10,000 years ago. As a consequence of plant diseases, world agriculture faces an estimated loss of 18% annually amounting to approximately 1,300 billion INR (Oerke, 2006). *Sclerotium rolfsii* Sacc. is a devastating soilborne fungus that infects more than five hundred agricultural and horticultural plant species around the world, causing root rot, stem rot, collar rot, willow, and foot rot diseases (Aycock, 1966; Punja, 1985). Crucifers, cucurbits, and legumes are its most common hosts. The fungus is of considerable economic significance because it causes 10–100 percent crop loss in different crops. Due to the formation of excessive sclerotia it may persist in soil for several years (Punja, 1985). Chemical crop protection measures continue to play an important role in agribusiness in spite of the emergence of novel biotechnological and biological solutions. Resistance to fungicides is becoming a major problem generating disease control problems in many crops. In the last decade, numerous cases of fungicide resistance have occurred worldwide, leading to loss of several fungicides (Hollomon, 2015). Therefore, the discovery of new molecules has assumed critical importance to combat the fungal infections.

Chromone is a group of naturally occurring compounds, reported, mainly in plants. The chromone moiety is a pharmacophore in a large number of natural and synthetic bioactive molecules. The chromone scaffold is present in plant’s secondary metabolites: flavones and isoflavones. Chromones are reported to have anti-tumor, anti-inflammatory and anti-fungal activities, and inhibitory activities, toward, phosphatases, kinases, cyclooxygenases, aromatases, acetylcholinesterases, and monoamine oxidases (Gasparova et al., 1997; Gaspar et al., 2011, Gaspar et al., 2014).

Our group has been actively involved in developing new crop protection products (Kaushik et al., 2019; Yadav et al., 2019). In our quest for biologically active molecules, we herein reported the synthesis of a series of iodochromones and their evaluation against *S. rolfsii*. A QSAR study was carried out with the objective to find the molecular properties which affect the fungicidal activity.

## Experimental

### Chemicals and Instruments

Chemicals were purchased from industrial manufacturers and, unless otherwise specified, were used without any further purification. Precoated Merck-silica gel 60F_254_ plates were used for thin layer chromatography (TLC); UV cabinet was used to detect developed plates. Column chromatography was performed with 100–200 mesh silica gels. Melting points were recorded by Buchi M-560 instrument and were uncorrected. The IR spectroscopy was done with PerkinElmer 2000 FT-IR spectrometer; KBr disc were used for samples preparation. The ^1^H NMR and ^13^C spectra were recorded on a Jeol alpha-400 and at 100.6 MHz, respectively, using TMS as an internal standard. The chemical shift values were on *δ* scale and the coupling constants (J) were in Hz. Signals from OH groups in ^1^HNMR spectra were verified by removing them by shaking in D_2_O.

High Resolution Mass Spectrometry (HRMS) was performed by AB SCIEX Triple TOFTM 5600+ equipped with Turboion Spray (TIS), SCIEX ExionLC, and PDA detector. Compounds were separated through C-18 column (2.7 µm, 4.6 × 100 mm) by eluting with methanol and water (98:2, v/v) at 0.3 ml/min at 40°C.

ED_50_ values were estimated with the SPSS statistical package. The whole computational work was carried out by using VLifeMDS QSAR plus 4.6 software using the Lenovo PC having window 8.1 operating system and Intel (R) Celeron (R) processor.

### Synthesis

#### Synthesis of Substituted 2-Hydroxyacetophenones (2a–2n)

2-hydroxyacetophenone and bromoalkanes or iodoalkanes of different chain lengths were taken in a molar ratio of 1:1.2 and stirred continuously for 6 h at 60°C in the presence of K_2_CO_3_ and acetone. Reaction was supervised by thin layer chromatography (TLC) with ethyl acetate: hexane (3:7).

#### General Method for Synthesis of Substituted 3-Dimethylamino-1-(2-hydroxyphenyl)propenones (3a–3u)

A mixture of substituted 2-hydroxyacetophenones (2a–2u) (1.2 mmol) and *N*,*N*-dimethylformamidedimethylacetal (2.4 mmol, 2 molequiv) was heated at 90°C overnight and allowed to cool. The reaction was worked up by removing solvent using vacuum evaporation on Heidolph rotary evaporator Hei-VAP, and the pure product were obtained by column chromatography with hexane: ethyl acetate (85:15). Compounds **3f, 3g, 3h, 3i, 3j, 3k, 3l, 3m, 3n,** and **3o** were reported for the first time in the literature.

### Spectral Analysis of Synthesized 3-Dimethylamino-1-(2-hydroxyphenyl)propenones (3a–3u)

#### 3-Dimethylamino-1-(2-hydroxyphenyl)propenone (3a)

It was obtained as a brown solid in 82% yield; m.p.:127–128°C, R_f_: 0.42 (ethyl acetate: hexane, 3:7). IR (cm^−1^): 3,710 (O-H stretch), 2,949 (aliphatic C-H), 1,635 (C=O), 1,565 (C=C stretch), 1,455 (C-H bending of CH_2_), and 1,264 (C-O). ^1^H NMR (400 MHz, CDCl_3_): *δ* 2.87 (3H, s, N-*CH*_*3*_), 3.12 (3H, s, N-*CH*_*3*_), 5.73 (1H, d, J = 12, H-3), 6.62–6.73 (4H, m, ArH), and 7.88 (1H, d, J = 12.4, H-2).^13^C NMR (100.6 MHz, CDCl_3_): 190.31 (C-1), 162.10 (C-2′), 154.05 (C-3), 133.97 (C-4″), 130.08 (C-6″), 122.31 (C-1″), 121.42.84 (C-5″), 116.21 (C-3″), and 89.75 (C-2). HR-MS for C_11_H_13_NO_2_ [M + H]^+^ *m/z:* Calcd 192.1091; Observed 192.1087. The major mass fragments observed were C_7_H_5_O_3_^+^ (137), C_5_H_8_O^+^ (98), and C_4_H_10_N^+^ (72).

#### 3-Dimethylamino-1-(2-hydroxy-4-methoxyphenyl)propenone (3b)

It was obtained as bright light yellow solid in 87% yield; m.p.: 137–140°C, R_f_: 0.42 (ethyl acetate: hexane, 3:7). IR (cm^−1^): 3,711 (O-H stretch), 2,953 (aliphatic C-H), 1,630 (C=O), 1,561 (C=C stretch), 1,458 (C-H bending of CH_2_), and 1,266 (C-O). ^1^HNMR (400 MHz, CDCl_3_): *δ* 2.95 (3H, s, N-*CH*_*3*_), 3.13 (3H, s, N-*CH*_*3*_), 3.98 (2H, t, J = 6.8, H-1′), 5.67 (1H, d, J = 12, H-2), 6.33–6.37 (2H, m, H-5′ and H-3′), 7.58 (1H, d, J = 8.8 H-6″), and 7.85 (1H, d, J = 12.4, H-3).^13^C NMR (100.6 MHz, CDCl_3_): 190.81 (C-1), 165.53 (^4^C″), 164.06 (^2^C″), 154.05 (C-3), 129.79 (C-6″), 113.72 (C-1″), 106.84 (C-5′), 101.53 (C-3″), and 89.75 (C-2). HR-MS for C_12_H_15_NO_3_ [M + H]^+^ *m/z*: Calcd 222.1124; Observed 222.1118. The major mass fragments observed were C_8_H_7_O_3_^+^(151), C_7_H_5_O_3_^+^(137), C_5_H_8_NO^+^(98), and C_4_H_10_N^+^(72).

#### 3-Dimethylamino-1-(2-hydroxy-4-ethoxyphenyl)propenone (3c)

It was obtained as bright light yellow solid in 85% yield; m.p.: 140–142°C, R_f_: 0.43 (ethyl acetate: hexane, 3:7). IR (cm^−1^): 3,708 (O-H stretch), 2,951 (aliphatic C-H), 1,642 (C=O), 1,563 (C=C stretch), 1,455 (C-H bending of CH_2_), and 1,258 (C-O). ^1^HNMR (400 MHz, CDCl_3_): *δ* 1.41 (3H, t, J = 6.8, H-2′), 4.07 (2H, q, H-1′), 2.95 (3H, s, N-*CH*_*3*_), 3.12 (3H, s, N-*CH*_*3*_), 3.98 (2H, t, J = 6.8, H-1′), 5.69 (1H, d, J = 9.6, H-2), 6.35–6.39 (2H, m, H-5″ and H-3″), 7.60 (1H, d, J = 8.8 H-6″), and 7.85 (1H, d, J = 9.6, H-3). ^13^C NMR (100.6 MHz, CDCl_3_): 190.83 (C-1), 165.56 (C-4″), 164.09 (C-2″), 154.07 (C-3), 129.80 (C-6″), 113.77 (C-1″), 106.86 (C-5″), 101.57 (C-3″), 89.78 (^2^C), 68.20 (C-1′), and 15.07 (C-2′). HR-MS for C_13_H_17_NO_3_ [M + H]^+^ *m/z:* Calcd 236.1281; Observed 236.1268. The major mass fragments observed were C_9_H_9_O_3_^+^(165), C_7_H_5_O_3_^+^(137), C_5_H_8_NO^+^(98), and C_4_H_10_N^+^(72).

#### 3-Dimethylamino-1-(2-hydroxy- 4-propoxyphenyl)propenone (3d)

It was obtained as orange yellow solid in 81% yield; m.p.: 121–124°C, R_f_: 0.45 (ethyl acetate: hexane, 3:7). IR (cm^−1^): 3,709 (O-H stretch), 2,949 (aliphatic C-H), 1,641 (C=O), 1,569 (C=C stretch), 1,461 (C-H bending of CH_2_), and 1,268 (C-O). ^1^HNMR (400 MHz, CDCl_3_): *δ* 1.06 (3H, t, J = 7.2, H-3′), 1.73–1.88 (2H, m, H-2′), 2.95 (3H, s, N-*CH*_*3*_), 3.12 (3H, s, N-*CH*_*3*_), 3.99 (2H, t, J = 6.8, H-1′), 5.69 (1H, d, J = 9.6, H-2), 6.35–6.39 (2H, m, H-5″ and H-3″), 7.62 (1H, d, J = 8.8 H-6″), and 7.84 (1H, d, J = 9.6, H-3).^13^C NMR (100.6 MHz, CDCl_3_): 190.81 (C-1), 165.53 (C-4″), 164.06 (C-2″), 154.05 (C-3), 129.81 (C-6″), 113.62 (C-1″), 106.65 (C-5″), 101.53 (C-3″), 89.75 (C-2), 68.18 (C-1′), 31.07 (C-2′), and 15.60 (C-3′). HR-MS for C_14_H_19_NO_3_ [M + H]^+^ *m/z*: Calcd 250.1437; Observed 250.1424. The major mass fragments observed were C_10_H_11_O_3_^+^(179), C_7_H_5_O_3_^+^(137), C_5_H_8_NO^+^(98), and C_4_H_10_N^+^(72).

#### 3-Dimethylamino-1-(2-hydroxy-4-isopropoxyphenyl)propenone (3e)

It was obtained as orange yellow solid in 77% yield; m.p.: 143–145°C, R_f_: 0.48 (ethyl acetate: hexane, 3:7). IR (cm^−1^): 3,740 (O-H stretch), 2,954 (aliphatic C-H), 1,647 (C=O), 1,563 (C=C stretch), 1,458 (C-H bending of CH_2_), and 1,263 (C-O). ^1^HNMR (400 MHz, CDCl_3_): *δ* 1.30 (1H, d, J = 4.8 Hz, CH_3_), 4.63–4.65 (1H, m, H-1′), 2.95 (3H, s, N-*CH*_*3*_), 3.12 (3H, s, N-*CH*_*3*_), 5.69 (1H, d, J = 12.4, H-2), 6.31–6.37 (2H, m, H-5″ and H-3″), 7.61 (1H, d, *J* = 8.8 H-6″), and 7.82 (1H, d, J = 12.4, H-3).^13^C NMR (100.6 MHz, CDCl_3_): 190.90 (C-1), 164.93 (C-4″), 164.08 (C-2″), 154.34 (C-3), 130.09 (C-6″), 114.31 (C-1″), 106.91 (C-5″), 101.75 (C-3″), 89.87 (C-2), 69.22 (C-1′), and 22.65 (*iC*H_3_). HR-MS for C_14_H_19_NO_3_ [M + H]^+^ *m/z:* Calcd 250.1437; Observed 250.1424. The major mass fragments observed were C_10_H_11_O_3_^+^(179), C_7_H_5_O_3_^+^(137), C_5_H_8_NO^+^(98), and C_4_H_10_N^+^(72).

#### 3-Dimethylamino-1-(4-Butoxy-2-hydroxyphenyl)propenone (3f)

It was obtained as lemon yellow solid in 88% yield; m.p.: 103–105°C R_f_: 0.51 (ethyl acetate: hexane, 3:7). IR (cm^−1^): 3,716 (O-H stretch), 2,955 (aliphatic C-H), 1,638 (C=O), 1,566 (C=C stretch), 1,465 (C-H bending of CH_2_), and 1,256 (C-O). ^1^HNMR (400 MHz, CDCl_3_): *δ* 0.96 (3H, t, J = 7.2, H-4′), 1.43–1.78 (2H, m, H-2′ and H-3′), 2.95 (3H, s, N-*CH*_*3*_), 3.16 (3H, s, N-*CH*_*3*_), 3.98 (2H, t, J = 6.8, H-1′), 5.63 (1H, d, J = 12, H-2), 6.32–6.38 (2H, m, H-5″ and H-3″), 7.55 (1H, d, J = 8.8 H-6″), and 7.83 (1H, d, J = 12.4, H-3).^13^C NMR (100.6 MHz, CDCl_3_): 190.79 (C-1), 165.49 (C-4″), 164.05 (C-2″), 154.01 (C-3), 129.76 (C-6″), 113.77 (C-1″), 106.80 (C-5″), 101.54 (C-3″), 89.85 (C-2), 68.13 (C-1′), 31.52 (C-2′), 25.70 (C-3′), and 14.29 (C-4′). HR-MS for C_15_H_21_NO_3_ [M + H]^+^ *m/z:* Calcd 264.1594; Observed 264.1585. The major mass fragments observed were C_11_H_13_O_3_^+^(193), C_7_H_5_O_3_^+^(137), C_5_H_8_NO^+^(98), and C_4_H_10_N^+^(72).

#### 3-Dimethylamino-1-(4-Pentyloxy-2-hydroxyphenyl)propenone (3g)

It was obtained as lemon yellow solid in 78% yield; m.p.: 98–100°C, R_f_: 0.53 (ethyl acetate: hexane, 3:7). IR (cm^−1^): 3,707 (O-H stretch), 2,943 (aliphatic C-H), 1,642 (C=O), 1,576 (C=C stretch), 1,468 (C-H bending of CH_2_), and 1,267 (C-O). ^1^HNMR (400 MHz, CDCl_3_): *δ* 0.91 (3H, t, *J* = 6.8, H-5′), 1.31–1.79 (6H, m, H-2′,H-3′and H-4′), 2.95 (3H, s, N-*CH*_*3*_), 3.15 (3H, s, N-*CH*_*3*_), 3.96 (2H, t, J = 6.8, H-1′), 5.66 (1H, d, J = 12.4, H-2), 6.34–6.40 (2H, m, H-5″ and H-3″), 7.59 (1H, d, J = 8.8 H-6″), and 7.84 (1H, d, J = 12.4, H-3). ^13^C NMR (100.6 MHz, CDCl_3_): 190.83 (C-1), 165.54 (C-4″), 164.01 (C-2″), 154.03 (C-3), 129.71 (C-6″), 113.70 (C-1″), 106.82 (C-5″), 101.51 (C-3″), 89.83 (C-2), 68.17 (C-1′), 31.50 (C-2′), 28.93 (C-3′), 25.66 (C-4′), and 14.20 (C-5′). HR-MS for C_16_H_23_NO_3_ [M + H]^+^ *m/z:* Calcd 278.1750; Observed 278.1765. The major mass fragments observed were C_12_H_15_O_3_^+^(207), C_7_H_5_O_3_^+^(137), C_5_H_8_NO^+^(98), and C_4_H_10_N^+^(72).

#### 3-Dimethylamino-1-(4-Hexyloxy-2-hydroxyphenyl)propenone (3h)

It was obtained as pale yellow solid in 75% yield; m.p.: 87–92°C, R_f_: 0.60 (ethyl acetate: hexane, 3:7). IR (cm^−1^): 3,718 (O-H stretch), 2,937 (aliphatic C-H), 1,639 (C=O), 1,569 (C=C stretch), 1,470 (C-H bending of CH_2_), and 1,264 (C-O). ^1^HNMR (400 MHz, CDCl_3_): *δ* 0.90 (3H, t, J = 6.8, H-6′), 1.30–1.79 (8H, m, H-2′, H-3′, H-4′ and H-5′), 2.95 (3H, s, N-*CH*_*3*_), 3.16 (3H, s, N-*CH*_*3*_), 3.98 (2H, t, J = 6.8, H-1′), 5.69 (1H, d, J = 12, H-2), 6.35–6.39 (2H, m, H-5″ and H-3″), 7.60 (1H, d, J = 8.8 H-6″), and 7.84 (1H, d, J = 12.4, H-3).^13^C NMR (100.6 MHz, CDCl_3_): 190.80 (C-1), 165.58 (C-4″), 164.03 (C-2″), 154.05 (C-3), 129.74 (C-6″), 113.73 (C-1″), 106.86 (C-5″), 101.53 (C-3″), 89.87 (C-2), 68.19 (C-1′), 31.57 (C-2′), 28.95 (C-3′), 25.67 (C-4′), 22.65 (C-5′), and 14.19 (C-6′). HR-MS for C_17_H_25_NO_3_ [M + H]^+^ *m/z:* Calcd 292.1907; Observed 292.1912. The major mass fragments observed were C_13_H_17_O_3_^+^(221), C_7_H_5_O_3_^+^(137), C_5_H_8_NO^+^(98), and C_4_H_10_N^+^(72).

#### 3-Dimethylamino-1-(4-Heptyloxy-2-hydroxyphenyl)propenone (3i)

It was obtained as pale yellow solid in 80% yield; m.p.: 90–92°C, R_f_: 0.50 (ethyl acetate: hexane, 3:7). IR (cm^−1^): 3,709 (O-H stretch), 2,941 (aliphatic C-H), 1,631 (C=O), 1,572 (C=C stretch), 1,472 (C-H bending of CH_2_), and 1,266 (C-O). ^1^HNMR (400 MHz, CDCl_3_): *δ* 0.88 (3H, t, J = 6.8, H-7′), 1.23–1.78 (10H, m, H-2′, H-3′, H-4′, H-5′and H-6′), 2.92 (3H, s, N-*CH*_*3*_), 3.14 (3H, s, N-*CH*_*3*_), 3.95 (2H, t, J = 6.8, H-1′), 5.66 (1H, d, J = 12, H-2), 6.35–6.39 (2H, m, H-5″ and H-3″), 7.55 (1H, d, J = 8.8 H-6″), and 7.82 (1H, d, J = 12.4, H-3). ^13^C NMR (100.6 MHz, CDCl_3_): 190.81 (C-1), 165.59 (C-4″), 164.05 (C-2″), 154.07 (C-3), 129.72 (C-6″), 113.74 (C-1″), 106.89 (C-5″), 101.54 (C-3″), 89.89 (C-2), 68.16 (C-1′), 31.57–22.66 (C-2′, C-3′, C-4′, C-5′, C-6′), and 14.20 (C-7′). HR-MS for C_18_H_27_NO_3_ [M + H]^+^ *m/z*: Calcd 306.2063; Observed 306.2059. The major mass fragments observed were C_14_H_19_O_3_^+^(235), C_7_H_5_O_3_^+^(137), C_5_H_8_O^+^(98), and C_4_H_10_N^+^(72).

#### 3-Dimethylamino-1-(2-hydroxy-4-octyloxyphenyl)propenone (3j)

It was obtained as yellow solid in 76% yield; m.p.: 70–72°C, R_f_: 0.45 (ethyl acetate: hexane, 3:7). IR (cm^−1^): 3,718 (O-H stretch), 2,936 (aliphatic C-H), 1,641 (C=O), 1,579 (C=C stretch), 1,475 (C-H bending of CH_2_), and 1,262 (C-O). ^1^HNMR (400 MHz, CDCl_3_): *δ* 0.90 (3H, t, J = 6.8, H-8′), 1.24–1.78 (12H, m, H-2′, H-3′, H-4′, H-5′, H-6′, and H-7′), 2.92 (3H, s, N-*CH*_*3*_), 3.13 (3H, s, N-*CH*_*3*_), 3.94 (2H, t, J = 6.8, H-1′), 5.67 (1H, d, J = 12, H-3), 6.35–6.39 (2H, m, H-5″ and H-3″), 7.58 (1H, d, J = 8.8 H-6″), and 7.82 (1H, d, J = 12.4, H-2).^13^C NMR (100.6 MHz, CDCl_3_): 190.80 (C-1), 165.57 (C-4″), 164.07 (C-2″), 154.04 (C-3), 129.74 (C-6″), 113.72 (C-1″), 106.90 (C-5″), 101.56 (C-3″), 89.90 (C-2), 68.15 (C-1′), 31.86–22.65 (C-2′, C-3′, C-4′, C-5′, C-6′, C-7′), and 14.46 (C-8′). HR-MS for C_19_H_29_NO_3_ [M + H]^+^ *m/z:* Calcd 320.2220; Observed 320.2214. The major mass fragments observed were C_15_H_21_O_3_^+^(249), C_7_H_5_O_3_^+^(137), C_5_H_8_NO^+^(98), and C_4_H_10_N^+^(72).

#### 3-Dimethylamino-1-(2-hydroxy-4-nonyloxyphenyl)propenone (3k)

It was obtained as yellow solid in 86% yield; m.p.: 78–80°C, R_f_: 0.49 (ethyl acetate: hexane, 3:7). IR (cm^−1^): 3,711 (O-H stretch), 2,945 (aliphatic C-H), 1,642 (C=O), 1,593 (C=C stretch), 1,478 (C-H bending of CH_2_), and 1,265 (C-O).^1^HNMR (400 MHz, CDCl_3_): *δ* 0.86 (3H, t, J = 6.8, H-9′), 1.26–1.78 (12H, m, H-2′, H-3′, H-4′, H-5′, H-6′, H-7′, and H-8′), 2.92 (3H, s, N-*CH*_*3*_), 3.13 (3H, s, N-*CH*_*3*_), 3.98 (2H, t, J = 6.8, H-1′), 5.64 (1H, d, J = 12, H-2), 6.35–6.39 (2H, m, H-5″ and H-3″), 7.59 (1H, d, J = 8.8 H-6″), and 7.87 (1H, d, J = 12.4, H-3). ^13^C NMR (100.6 MHz, CDCl_3_): 190.77 (C-1), 165.56 (C-4″), 164.03 (C-2″), 154.01 (C-3), 129.75 (C-6″), 113.69 (C-1″), 106.91 (C-5″), 101.55 (C-3″), 89.86 (C-2), 68.14 (C-1′), 31.88–22.63 (C-2′, C-3′, C-4′, C-5′, C-6′, C-7′, C-8′), and 14.45 (C-9′). HR-MS for C_20_H_31_NO_3_ [M + H]^+^ *m/z*: Calcd 334.2376; Observed 334.2382. The major mass fragments observed were C_16_H_23_O_3_^+^(263), C_7_H_5_O_3_^+^(137), C_5_H_8_NO^+^(98), and C_4_H_10_N^+^(72).

#### 3-Dimethylamino 1-(4-decyloxy-2-hydroxyphenyl)propenone (3l)

It was obtained as yellow solid in 83% yield; m.p.:76–79°C, R_f_: 0.54 (ethyl acetate: hexane, 3:7). IR (cm^−1^): 3,708 (O-H stretch), 2,955 (aliphatic C-H), 1,629 (C=O), 1,586 (C=C stretch), 1,498 (C-H bending of CH_2_), and 1,272 (C-O). ^1^HNMR (400 MHz, CDCl_3_): *δ* 0.86 (3H, t, J = 6.8, H-10′), 1.25–1.78 (16H, m, H-2′, H-3′, H-4′, H-5′, H-6′, H-7′, H-8′, and H-9′), 2.92 (3H, s, N-*CH*_*3*_), 3.13 (3H, s, N-*CH*_*3*_), 3.94 (2H, t, J = 6.8 H-1′) 5.68 (1H, d, J = 12, H-2), 6.33–6.37 (2H, m, H-5″ and H-3″) 7.56 (1H, d, J = 8.8 H-6″), and 7.83 (1H, d, J = 12.4, H-3).^13^C NMR (100.6 MHz, CDCl_3_): 190.81 (C-1), 165.59 (C-4″), 164.05 (C-2″), 154.03 (C-3), 129.73 (C-6″), 113.74 (C-1″), 106.93 (C-5″), 101.58 (C-3″), 89.89 (C-2), 68.18 (C-1′), 31.97–22.76 (C-3′, C4′, C5′, C-6′, C-7′, C-8′, C-9′), and 14.19 (C-10′). HR-MS for C_21_H_33_NO_3_ [M + H]^+^ *m/z*: Calcd 348.2533; Observed 348.2550. The major mass fragments observed were C_17_H_25_O_3_^+^(277), C_7_H_5_O_3_^+^(137), C_5_H_8_NO^+^(98), and C_4_H_10_N^+^(72).

#### 3-Dimethylamino 1-(4-dodecyloxy-2-hydroxyphenyl)propenone (3m)

It was obtained as yellow solid in 81% yield; m.p.: 86–88°C, R_f_: 0.51 (ethyl acetate: hexane, 3:7). IR (cm^−1^): 3,757 (O-H stretch), 2,945 (aliphatic C-H), 1,634 (C=O), 1,539 (C=C stretch), 1,495 (C-H bending of CH_2_), and 1,278 (C-O). ^1^HNMR (400 MHz, CDCl_3_): *δ* 0.87 (3H, t, J = 6.8, H-12′), 1.26–1.77 (20H, m, H-2′, H-3′, H-4′, H-5′, H-6′, H-7′, H-8′, H-9′, H-10′, and H-11′), 2.93 (3H, s, N-*CH*_*3*_), 3.15 (3H, s, N-*CH*_*3*_), 3.94 (2H, t, J = 6.8, H-1′), 5.69 (1H, d, J = 12, H-2), 6.33–6.37 (2H, m, H-5″ and H-3″) 7.58 (1H, d, J = 7.2, H-6″), and 7.84 (1H, d, J = 12.4, H-3). ^13^C NMR (100.6 MHz, CDCl_3_): 190.67 (C-1), 165.59 (C-4″), 164.04 (C-2″), 154.03 (C-3), 129.74 (C-6″), 113.73 (C-1″), 106.93 (C-5″), 101.57 (C-3″), 89.89 (C-2), 68.18 (C-1′), 31.94–22.73 (C-3′, C-4′, C-5′, C-6′, C-7′, C-8′, C-9′, C-10′, C-11′), and 14.20 (C-12′). HR-MS for C_23_H_37_NO_3_ [M + H]^+^ *m/z*: Calcd 376.2846; Observed 376.2858. The major mass fragments observed were C_19_H_29_O_3_^+^(305), C_7_H_5_O_3_^+^(137), C_5_H_8_O^+^(98), and C_4_H_10_N^+^(72).

#### 3-Dimethylamino 1-(4-tridecyloxy-2-hydroxyphenyl)propenone (3n)

It was obtained as yellow solid in 72% yield; m.p.: 87–90°C, R_f_: 0.49 (ethyl acetate: hexane, 3:7). IR (cm^−1^): 3,770 (O-H stretch), 2,941 (aliphatic C-H), 1,630 (C=O), 1,545 (C=C stretch), 1,497 (C-H bending of CH_2_), and 1,287 (C-O). ^1^HNMR (400 MHz, CDCl_3_): *δ* 0.86 (3H, t, J = 6.8, H-13′), 1.24–1.79 (22H, m, H-2′, H-3′, H-4′, H-5′, H-6′, H-7′, H-8′, H-9′, H-10′, H-11′, and H-12′), 2.92 (3H, s, N-*CH*_*3*_), 3.14 (3H, s, N-*CH*_*3*_), 3.98 (2H, t, J = 6.8, H-1′), 5.66 (1H, d, J = 12, H-2), 6.32–6.35 (2H, m, H-5″ and H-3″), 7.56 (1H, d, J = 8.8, H-6″), and 7.80 (1H, d, J = 12.4, H-3). ^13^C NMR (100.6 MHz, CDCl_3_): 190.69 (C-1), 165.60 (C-4″), 164.05 (C-2″), 154.02 (C-3), 129.73 (C-6″), 113.74 (C-1″), 106.94 (C-5″), 101.58 (C-3″), 89.90 (C-2), 68.18 (C-1′), 32–22.77 (C-3′, C4′, C5′, C-6′, C-7′, C-8′, C-9′, C-10′, C-11′, C-12′), and 14.19 (C-13′). HR-MS for C_24_H_39_NO_3_ [M + H]^+^ *m/z*: Calcd 390.3002; Observed 390.3010. The major mass fragments observed were C_20_H_31_O_3_^+^(319), C_7_H_5_O_3_^+^(137), C_5_H_8_NO^+^(98), and C_4_H_10_N^+^(72).

#### 3-Dimethylamino 1-(4-tetradecyloxy-2-hydroxyphenyl)propenone (3o)

It was obtained as yellow solid in 78% yield; m.p.: 76–81°C, R_f_: 0.50 (ethyl acetate: hexane, 3:7). IR (cm^−1^): 3,763 (O-H stretch), 2,934 (aliphatic C-H), 1,645 (C=O), 1,536 (C=C stretch), 1,489 (C-H bending of CH_2_), and 1,278 (C-O). ^1^HNMR (400 MHz, CDCl_3_): *δ* 0.87 (3H, t, J = 6.8, H-14′), 1.26–1.80 (24H, m, H-2′, H-3′, H-4′, H-5′, H-6′, H-7′, H-8′, H-9′, H-10′, H-11′ H-12′, and H-13′), 2.92 (3H, s, N-*CH*_*3*_), 3.19 (3H, s, N-*CH*_*3*_), 3.99 (2H, t, J = 6.8, H-1′), 5.69 (1H, d, J = 12, H-2), 6.31–6.36 (2H, m, H-5″ and H-3″), 7.55 (1H, d, J = 8.8, H-6″), and 7.83 (1H, d, J = 12.4, H-3). ^13^C NMR (100.6 MHz, CDCl_3_): 190.71 (C-1), 165.57 (C-4″), 164.03 (C-2″), 154.08 (C-3), 129.71 (C-6″), 113.70 (C-1″), 106.92 (C-5″), 101.55 (C-3″), 89.93 (C-2), 68.15 (C-1′), 31.97–22.71 (C-3′, C4′, C5′, C-6′, C-7′, C-8′, C-9′, C-10′, C-11′, C-12′, C-13′), and 14.16 (C-14′). HR-MS for C_25_H_41_NO_3_ [M + H]^+^ *m/z*: Calcd 404.3159; Observed 404.3143. The major mass fragments observed were C_21_H_33_O_3_^+^(333), C_7_H_5_O_3_^+^(137), C_5_H_8_NO^+^(98), and C_4_H_10_N^+^(72).

#### 3-Dimethylamino 1-(5-bromo-2-hydroxyphenyl)propenone (3p)

It was obtained as bright yellow solid in 87% yield; m.p.: 128–132°C, R_f_: 0.50 (ethyl acetate: hexane, 3:7). IR (cm^−1^): 3,735 (O-H stretch), 2,923 (aliphatic C-H), 1,638 (C=O), 1,548 (C=C stretch), 1,437 (C-H bending of CH_2_), and 1,258 (C-O). ^1^HNMR (400 MHz, CDCl_3_): *δ* 2.95 (3H, s, N-*CH*_*3*_), 3.18 (3H, s, N-*CH*_*3*_), 5.68 (1H, d, J = 9.6, H-2), 7.06 (1H, d, J = 7.2 Hz, H-3″) 7.43–7.47 (1H, m, H-4″), 7.78 (1H, d, J = 2, H-6″), and 7.90 (1H, d, J = 9.6, H-3). ^13^C NMR (100.6 MHz, CDCl_3_): 190.75 (C-1), 164.10 (C-2′), 154.06 (C-3), 134.44 (C-4′), 129.56 (C-6′), 124.63 (C-1′), 123.20 (C-3′), 118.73 (C-5′), and 89.93 (C-2). HR-MS for C_11_H_11_BrNO_2_ [M + H]^+^ *m/z*: Calcd 269.0045; Observed 269.0029. The major mass fragments observed were C_7_H_3_BrO_2_^+^(197), C_5_H_8_NO^+^(98), and C_4_H_10_N^+^(72).

#### 3-Dimethylamino-1-(5-chloro-2-hydroxyphenyl)propenone (3q)

It was obtained as bright yellow solid in 78% yield; m.p.: 125–128°C, R_f_: 0.53 (ethyl acetate: hexane, 3:7). IR (cm^−1^): 3,739 (O-H stretch), 2,925 (aliphatic C-H), 1,644 (C=O), 1,555 (C=C stretch), 1,429 (C-H bending of CH_2_), and 1,258 (C-O). ^1^HNMR (400 MHz, CDCl_3_): *δ* 2.93 (3H, s, N-*CH*_*3*_), 3.19 (3H, s, N-*CH*_*3*_), 5.69 (1H, d, J = 9.6, H-2), 6.81 (1H, d, J *=* 7.2 Hz, H-3″), 7.01–7.05 (1H, m, H-4″), 7.69 (1H, d, J = 2, H-6″), and 7.84 (1H, d, J = 9.6, H-3). ^13^C NMR (100.6 MHz, CDCl_3_): 190.63 (C-1), 158.67 (C-2), 154.19 (C-2″), 134.45 (C-4″), 131.26 (C-6″), 130.55 (C-5″), 121.10 (C-1″), 119.25 (C-3″), and 86.91 (C-3). HR-MS for C_12_H_13_ClNO_2_ [M + H]^+^ *m/z*: Calcd 239.0707; Observed 239.0712. The major mass fragments observed were C_7_H_3_ClO_2_^+^(153), C_5_H_8_NO^+^(98), and C_4_H_10_N^+^(72).

#### 3-Dimethylamino 1-(3,5-dichloro-2-hydroxyphenyl)propenone (3r)

It was obtained as bright yellow solid in 82% yield; m.p.: 137–141°C, R_f_: 0.48 (ethyl acetate: hexane, 3:7). IR (cm^−1^): 3,730 (O-H stretch), 2,918 (aliphatic C-H), 1,628 (C=O), 1,539 (C=C stretch), 1,422 (C-H bending of CH_2_), and 1,266 (C-O). ^1^HNMR (400 MHz, CDCl_3_): *δ* 2.99 (3H, s, N-*CH*_*3*_), 3.21 (3H, s, N-*CH*_*3*_), 5.64 (1H, d, J = 9.6, H-2), 7.41 (1H, d, J = 2, H-4″) 7.53 (1H, d, J = 2, H-6″), and 7.92 (1H, d, J = 9.6, H-3). ^13^C NMR (100.6 MHz, CDCl_3_): 190.66 (C-1), 164.10 (C-2″), 154.06 (C-3), 134.44 (C-4″), 130.60 (C-6″), 126.63 (C-5″), 122.20 (C-1″), 119.67 (C-3″), and 89.93 (C-2). HR-MS for C_11_H_11_Cl_2_NO_2_ [M + H]^+^ *m/z*: Calcd 260.0239; Observed 260.0224. The major mass fragments observed were C_7_H_3_Cl_2_O_2_^+^(188), C_5_H_8_NO^+^(98), and C_4_H_10_N^+^(72).

#### 3-Dimethylamino-1-(2-hydroxy-5-methylphenyl)propenone (3s)

It was obtained as bright yellow solid in 84% yield; m.p.: 105–109°C, R_f_: 0.52 (ethyl acetate: hexane, 3:7). IR (cm^−1^): 3,742 (O-H stretch), 2,922 (aliphatic C-H), 1,639 (C=O), 1,550 (C=C stretch), 1,431 (C-H bending of CH_2_), and 1,253 (C-O). ^1^HNMR (400 MHz, CDCl_3_): *δ* 2.97 (3H, s, N-*CH*_*3*_), 3.18 (3H, s, N-*CH*_*3*_), 5.61 (1H, d, J = 9.6, H-2), 6.27(1H, d, J *=* 7.2 Hz, H-3″) 7.01–7.05 (1H, m, H-4″), 7.81 (1H, d, J = 2, H-6″), and 7.89 (1H, d, J = 9.6, H-3). ^13^C NMR (100.6 MHz, CDCl_3_): 190.75 (C-1), 164.10 (C-2″), 154.06 (C-3), 135.20 (C-4″), 130.46 (C-6″), 129.54 (C-5″), 124.10 (C-1″), 119.12 (C-3″), and 89.93 (C-2). HR-MS for C_12_H_15_NO_2_ [M + H]^+^ *m/z*: Calcd 206.1175; Observed 206.1169. The major mass fragments observed were C_8_H_7_O_2_^+^(133), C_5_H_8_NO^+^(98), and C_4_H_10_N^+^(72).

#### 3-Dimethylamino-1-(5-fluoro-2-hydroxyphenyl)propenone (3t)

It was obtained as pale yellow solid in 88% yield; m.p.: 140–145°C, R_f_: 0.45 (ethyl acetate: hexane, 3:7). IR (cm^−1^): 3,740 (O-H stretch), 2,928 (aliphatic C-H), 1,641 (C=O), 1,557 (C=C stretch), 1,435 (C-H bending of CH_2_), and 1,258 (C-O). ^1^HNMR (400 MHz, CDCl_3_): *δ* 2.96 (3H, s, N-*CH*_*3*_), 3.15 (3H, s, N-*CH*_*3*_), 5.57 (1H, d, J = 9.6, H-2), 6.29 (1H, d, *J* = 7.2 Hz, H-3″) 7.03–7.07 (1H, m, H-4″), 7.82 (1H, d, J = 2, H-6″), and 7.87 (1H, d, J = 9.6, H-3). ^13^C NMR (100.6 MHz, CDCl_3_): 190.63 (C-1), 161.047 (C-5″), 156.13 (C-2″), 154.32 (C-2), 127.05 (C-1″), 121.10 (C-4″), 117.23 (C-6″), 115.04 (C-3′), and 89.83 (C-2). HR-MS for C_11_H_12_FNO_2_ [M + H]^+^ *m/z*: Calcd 210.0924; Observed 210.0920. The major mass fragments observed were C_21_H_33_O_3_^+^(333), C_7_H_4_FO_2_^+^(139), C_6_H_4_FO^+^(111), C_5_H_8_NO^+^(98), and C_4_H_10_N^+^(72).

#### 3-Dimethylamino-1-(5-chloro-2-hydroxy-4-methyl-phenyl)propenone (3u)

It was obtained as bright yellow solid in 80% yield; m.p.: 131–135°C, R_f_: 0.49 (ethyl acetate: hexane, 3:7). IR (cm^−1^): 3,742 (O-H stretch), 2,922 (aliphatic C-H), 1,639 (C=O), 1,550 (C=C stretch), 1,431 (C-H bending of CH_2_), and 1,253 (C-O).^1^HNMR (400 MHz, CDCl_3_): *δ* 2.41 (3H, CH_3_), 2.96 (3H, s, N-*CH*_*3*_), 3.10 (3H, s, N-*CH*_*3*_), 5.65 (1H, d, J = 9.6, H-2), 6.38 (1H, d, J = 7.2 Hz, H-3″),7.71 (1H, d, J = 2, H-6″), and 7.87 (1H, d, J = 9.6, H-3). ^13^C NMR (100.6 MHz, CDCl_3_): 190.71 (C-1), 158.67 (C-2), 155.15 (C-2″), 144.33 (C-4″), 131.53 (C-6″), 129.31 (C-5″), 122.10 (C-1′), 117.32 (C-3″), and 86.87 (C-3). HR-MS for C_12_H_14_ClNO_2_ [M + H]^+^ *m/z*: Calcd 240.0785; Observed 240.0779. The major mass fragments observed were C_8_H_7_ClO_2_^+^(169), C_5_H_8_NO^+^(98), and C_4_H_10_N^+^(72).

### General Method for the Synthesis of 3-Iodochromones (4a–4u)

**SCHEME 1:**
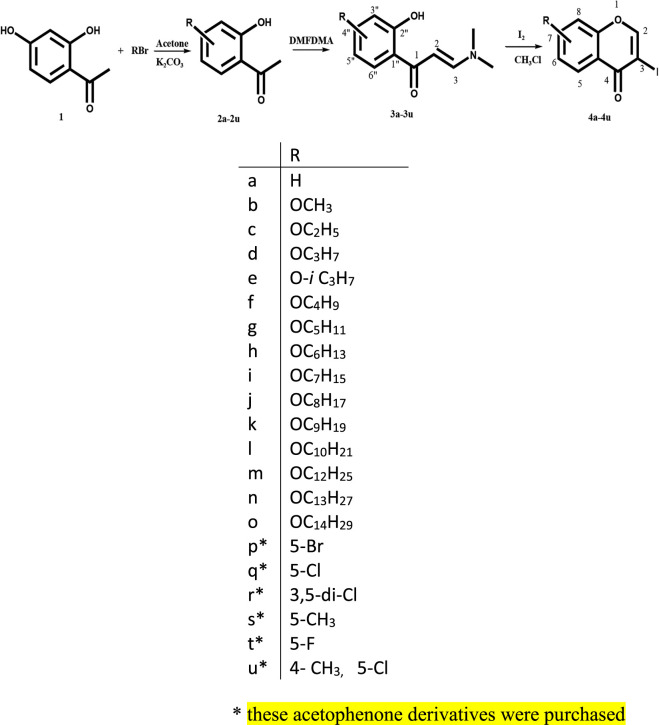
Synthesis of 3-iodochromones (Gammill, 1979).

The chromones were synthesized by taking substituted 3-(dimethylamino)-1-(2-hydroxyphenyl) propenone (0.54 mmol) in CHCl_3_ (15 ml) and iodine (1.09 mmol, 2 mol equiv), followed by stirring the mixture at 25°C for 8 h (Scheme 1) (Gammill, 1979). The formation of products was confirmed by thin-layer chromatography (TLC) in ethyl acetate:hexane (3:7) solvent system. The solution was washed with saturated Na_2_S_2_O_3_ (15 ml), and the aqueous layer was extracted with CHCl_3_ (20 ml). The solvent was removed using Heidolph rotary evaporator Hei-VAP. The synthesized chromones were purified by column chromatography to afford the pure chromones in 67–89% yield. Compounds **4f, 4g, 4h, 4i, 4j, 4k, 4l, 4m, 4n,** and **4o** are reported for the first time in the literature.

#### 3-Iodochromone (4a)

It was obtained as a white solid in 79% yield; m.p.: 102–103°C; R_f_: 0.49 (ethyl acetate: hexane, 3:7). IR (cm^−1^): 3,071 (aromatic C-H stretch), 2,932 (aliphatic C-H stretch), 1,646 (C=O stretch), 1,617(aromatic C=C stretch), 1,561 (pyrone ring C=C stretch), 1,426 (C-H bending of CH_2_), and 1,260 (C-O Stretch). ^1^H NMR (400 MHz, CDCl_3_): *δ* 6.83 (1H, d, J = 2.4 Hz, H-8), 6.93–6.98 (1H, m, H-6, H-7), 8.08 (1H, d, J = 8.8 Hz, H-5), and 8.17 (1H, s, H-2). ^13^C NMR (100.6 MHz, CDCl_3_): 175.44 (C-4), 158.93 (C-2), 155.99 (C-9), 134.11 (C-7), 126.32 (C-5), 123.70 (C-10), 118.30 (C-6), 101.33 (C-8), and 94.78 (C-1). HR-MS for C_9_H_5_IO_2_ [M + H]^+^ *m/z*: Calcd 272.9407; Observed 272.9406. The major mass fragment observed was C_9_H_6_IO_3_^+^ (288).

#### 3-Iodo-7-methoxychromone (4b)

It was obtained as white solid in 89% yield; m.p.: 140–145°C, R_f_: 0.51 (ethyl acetate: hexane, 3:7). IR (cm^−1^): 3,068 (aromatic C-H stretch), 2,934 (aliphatic C-H stretch), 1,642 (C=O stretch), 1,612 (aromatic C=C stretch), 1,559 (pyrone ring C=C stretch), 1,434 (C-H bending of CH_2_), and 1,258 (C-O Stretch). ^1^H NMR (400 MHz, CDCl_3_): *δ* 3.87 (3H, s, *OCH*_*3*_), 6.80 (1H, d, J = 2.4 Hz, H-8), 6.95–6.97 (1H, m, H-6), 8.10 (1H, d, J = 8.8 Hz, H-5), and 8.18 (1H, s, H-2). ^13^C NMR (100.6 MHz, CDCl_3_): 174.72 (C-4), 164.36 (C-7), 158.03 (C-2), 157.27 (C-9), 127.71 (C-5), 117.53 (C-6), 115.11 (C-10), 100.51 (C-8), and 55.99 (*OCH*_*3*_). HR-MS for C_10_H_7_IO_3_ [M + H]^+^ *m/z:* Calcd 302.9512; Observed 302.9518. The major mass fragment observed was C_9_H_6_IO_3_^+^ (288).

#### Ethoxy-3-Iodochromone (4c)

It was obtained as white solid in 82% yield; m.p.: 100–105°C, R_f_: 0.47 (ethyl acetate: hexane, 3:7). IR (cm^−1^): 3,065 (aromatic C-H stretch), 2,946 (aliphatic C-H stretch), 1,640 (C=O stretch), 1,617 (aromatic C=C stretch), 1,565 (pyrone ring C=C stretch), 1,436 (C-H bending of CH_2_), and 1,266 (C-O Stretch). ^1^H NMR (400 MHz, CDCl_3_): *δ* 1.42 (3H, t, J = 6.8 Hz, H-2′), 4.04 (2H, q, J = 6.8 Hz, H-1′), 6.29 (1H, d, J = 2.4 Hz, H-8), 6.34–6.36 (1H, m, H-6), 7.82 (1H, d, J = 8.8 Hz, H-5), and 7.93(1H, s, H-2). ^13^C NMR (100.6 MHz, CDCl_3_): 173.13 (C-4), 165.53 (C-7), 163.56 (C-2), 162.82 (C-9), 130.79 (C-5), 113.84 (C-10), 106.64 (C-6), 101.43 (C-8), 89.49 (C-3), 63.79 (C-1′), and 15.00 (C-2′). HR-MS for C_11_H_9_IO_3_ [M + H]^+^ *m/z*: Calcd 316.9669; Observed 316.9652. The major mass fragment observed was C_9_H_6_IO_3_^+^(288).

#### 3-Iodo-7-propoxychromone (4d)

It was obtained as yellowish white solid in 79% yield; m.p.: 88–91°C, R_f_: 0.46 (ethyl acetate: hexane, 3:7). IR (cm^−1^): 3,071 (aromatic C-H stretch), 2,944 (aliphatic C-H stretch), 1,642 (C=O stretch), 1,614 (aromatic C=C stretch), 1,559 (pyrone ring C=C stretch), 1,443 (C-H bending of CH_2_), and 1,256 (C-O Stretch). ^1^H NMR (400 MHz, CDCl_3_): *δ* 1.06 (3H, t, J = 7.2, H-3′), 1.73–1.88 (2H, m, H-2′), 3.99 (2H, t, J = 6.4 Hz, H-1′), 6.79 (1H, d, J = 2.4 Hz, H-8), 6.95–6.98 (1H, m, H-6), 8.11 (1H, d, J = 9.2 Hz, H-5), and 8.18 (1H, s, H-2). ^13^C NMR (100.6 MHz, CDCl_3_): 172.56 (C-4), 164.41 (C-7), 163.56 (C-2), 162.78 (C-9), 131.48 (C-5), 113.55 (C-10), 106.37 (C-6), 101.25 (C-8), 89.51 (C-3), 63.71 (C-1′), 31.42 (C-2′), and 14.98 (C-3′). HR-MS for C_12_H_11_IO_3_ [M + H]^+^ *m/z*: Calcd 330.9825; Observed 330.9820. The major mass fragment observed was C_9_H_6_IO_3_^+^(288).

#### 3-Iodo-7-isopropoxychromone (4e)

It was obtained as yellowish white solid in 76% yield; m.p.: 67–70°C, R_f_: 0.50 (ethyl acetate: hexane, 3:7). IR (cm^−1^): 3,061 (aromatic C-H stretch), 2,956 (aliphatic C-H stretch), 1,639 (C=O stretch), 1,612 (aromatic C=C stretch), 1,566 (pyrone ring C=C stretch), 1,453 (C-H bending of CH_2_), 1,265 and (C-O Stretch). ^1^H NMR (400 MHz, CDCl_3_): *δ* 1.31 (1H, d, J = 4.8 Hz, CH_3_), 4.66–4.68 (1H, m, H-1′), 6.83 (1H, d, J = 2 Hz, H-8), 6.96–6.98 (1H, m, H-6), 8.38 (1H, s, H-2), 8.15 (1H, d, J = 7.2 Hz, H-5), and 8.22 (1H, s, H-2). ^13^C NMR (100.6 MHz, CDCl_3_): 172.61 (C-4), 163.82 (C-7), 157.99 (C-9), 157.32 (C-2), 127.97 (C-5), 116.32 (C-6), 115.27 (C-10), 101.43 (C-8), 87.10 (C-3), 71.02 (C-1′), and 21.84 (isopropyl CH_3_). HR-MS for C_12_H_11_IO_3_ [M + H]^+^ *m/z*: Calcd 330.9825; Observed 330.9817. The major mass fragment observed was C_9_H_6_IO_3_^+^(288).

#### 7-Butoxy-3-iodochromone (4f)

It was obtained as white solid in 81% yield; m.p.: 86–88°C, R_f_: 0.57 (ethyl acetate: hexane, 3:7). IR (cm^−1^): 3,059 (aromatic C-H stretch), 2,945 (aliphatic C-H stretch), 1,643 (C=O stretch), 1,607 (aromatic C=C stretch), 1,575 (pyrone ring C=C stretch), 1,493 (C-H bending of CH_2_), and 1,247 (C-O Stretch). ^1^H NMR (400 MHz, CDCl_3_): *δ* 0.99 (3H, t, J = 6, H-4′), 1.47–1.81 (4H, m, H-2′ and 3′), 4.04 (2H, t, J = 5.2 Hz, H-1′), 6.80 (1H, d, J = 2 Hz, H-8), 6.95–6.98 (1H, m, H-6), 8.02 (1H, d, J = 7.2 Hz, H-5), and 8.19 (1H, s, H-2). ^13^C NMR (100.6 MHz, CDCl_3_): 172.42 (C-4), 163.82 (C-7), 158.99 (C-2), 157.97 (C-9), 127.48 (C-5), 116.07 (C-6), 115.30 (C-10), 101.33 (C-8), 87.53 (C-3), 68.78 (C-1′), 30.95 (C-2′), 19.18 (C-3′), and 14.16 (C-4′). HR-MS for C_13_H_13_IO_3_ [M + H]^+^ *m/z*: Calcd 344.9982; Observed 344.9974. The major mass fragment observed was C_9_H_6_IO_3_^+^(288).

#### 3-iodo-7-pentyloxychromone (4g)

It was obtained as yellowish white solid in 86% yield; m.p.: 77–80°C, R_f_: 0.52 (ethyl acetate: hexane, 3:7). IR (cm^−1^): 3,059 (aromatic C-H stretch), 2,922 (aliphatic C-H stretch), 1,637 (C=O stretch), 1,603 (aromatic C=C stretch), 1,570 (pyrone ring C=C stretch), 1,471 (C-H bending of CH_2_), and 1,253 (C-O Stretch). ^1^H NMR (400 MHz, CDCl_3_): *δ* 0.93 (3H, t, J = 6.8, H-5′), 1.36–1.83 (6H, m, H-2′, H-3′, and H-4′), 4.02 (2H, t, *J* = 6.4 Hz, H-1′), 6.79 (1H, d, J = 2.4 Hz, H-8), 6.95–6.97 (1H, m, H-6), 8.11 (1H, d, J = 8.8 Hz, H-5), and 8.18 (1H, s, H-2). ^13^C NMR (100.6 MHz, CDCl_3_): 172.70 (C-4), 164.05 (C-7), 158.04 (C-9), 157.26 (C-2), 128.05 (C-5), 115.71 (C-6), 115.59 (C-10), 100.54 (C-8), 87.20 (C-3), 68.93 (C-1′), 31.55 (C-2′), 28.93 (C-3′), 25.67 (C-4′), and 14.19 (C-5′). HR-MS for C_14_H_15_IO_3_ [M + H]^+^ *m/z*: Calcd 359.0138; Observed 359.0152. The major mass fragment observed was C_9_H_6_IO_3_^+^(288).

#### 7-Hexyloxy-3-iodochromone (4h)

It was obtained as white solid in 81% yield; m.p.: 88–90°C, R_f_: 0.48 (ethyl acetate: hexane, 3:7). IR (cm^−1^): 3,063 (aromatic C-H stretch), 2,919 (aliphatic C-H stretch), 1,635 (C=O stretch), 1,623 (aromatic C=C stretch), 1,571 (pyrone ring C=C stretch), 1,478 (C-H bending of CH_2_), and 1,257 (C-O Stretch). ^1^H NMR (400 MHz, CDCl_3_): 1.42 (3H, t, J = 6.8 Hz), 4.06 (2H, q, J = 6.8 Hz), 6.74 (1H, s), 6.90–6.93 (1H, m), 8.05 (1H, d, J = 9.2 Hz), and 8.17 (1H, s). ^13^C NMR (100.6 MHz, CDCl_3_): 172.70 (C-4), 163.98 (C-7), 158.03 (C-9), 157.27 (C-2), 128.04 (C-5), 115.73 (C-6), 115.56 (C-10), 100.53 (C-8), 87.20 (C-3), 68.92 (C-1′), 31.57 (C-2′), 28.96 (C-3′), 25.69 (C-4′), 22.65 (C-5′), and 14.20 (C-6′). HR-MS for C_15_H_17_IO_3_ [M + H]^+^ *m/z*: Calcd 373.0295; Observed 373.0241. The major mass fragment observed was C_9_H_6_IO_3_^+^(288).

#### 7-Heptyloxy-3-iodochromone (4i)

It was obtained as white solid in 79% yield; m.p.: 73–76°C, R_f_: 0.56 (ethyl acetate: hexane, 3:7). IR (cm^−1^): 3,083 (aromatic C-H stretch), 2,921 (aliphatic C-H stretch), 1,645 (C=O stretch), 1,612 (aromatic C=C stretch), 1,577 (pyrone ring C=C stretch), 1,463 (C-H bending of CH_2_), and 1,259 (C-O Stretch). ^1^H NMR (400 MHz, CDCl_3_): *δ* 0.89 (3H, t, J = 6.8, H-7′), 1.27–1.82 (10H, m, H-2′, H-3′, H-4′, H-5′, H-6′), 4.02 (2H, t, J = 6.8 Hz, H-1′), 6.79 (1H, d, J = 2.4 Hz, H-8), 6.95–6.98 (1H, m, H-6), 8.12 (1H, d, J = 8.8 Hz, H-5),and 8.18 (1H, s, H-2). ^13^C NMR (100.6 MHz, CDCl_3_): 172.70 (C-4), 163.99 (C-7), 158.05 (C-9), 157.27 (C-2), 128.05 (C-5), 115.72 (C-6), 115.59 (C-10), 100.54 (C-8), 87.20 (C-3), 68.93 (C-1′), 31.57–22.67 (C-2′, C-3′, C-4′, C-5′,C-6′), and 14.19 (C-7′). HR-MS for C_16_H_19_IO_3_ [M + H]^+^ *m/z*: Calcd 387.0451; Observed 387.0446. The major mass fragment observed was C_9_H_6_IO_3_^+^(288).

#### 3-Iodo-7-Octyloxychromone (4j)

It was obtained as yellowish white solid in 76% yield; m.p.: 73–74°C, R_f_: 0.49 (ethyl acetate: hexane, 3:7). IR (cm^−1^): 3,045 (aromatic C-H stretch), 2,923 (aliphatic C-H stretch), 1,642 (C=O stretch), 1,615 (aromatic C=C stretch), 1,593 (pyrone ring C=C stretch), 1,468 (C-H bending of CH_2_), and 1,266 (C-O Stretch). ^1^H NMR (400 MHz, CDCl_3_): *δ* 0.87 (3H, t, J = 6.8, H-8′), 1.24–1.84 (12H, m, H-2′, H-3′, H-4′, H-5′, H-6′, and H-7′), 4.03 (2H, t, J = 6.8 Hz, H-1′), 6.79 (1H, d, J = 2 Hz, H-8), 6.95–6.98 (1H, m, H-6), 8.12 (1H, d, J = 8.8 Hz, H-5), and 8.18 (1H, s, H-2). ^13^C NMR (100.6 MHz, CDCl_3_): 172.46 (C-4), 163.87 (C-7), 159.07 (C-2), 158.05 (C-9), 127.54 (C-5), 116.12 (C-6), 115.36 (C-10), 101.40 (C-8), 87.55 (C-3), 69.06 (C-1′), 31.86–22.66 (C-2′, C-3′, C-4′, C-5′, C-6′, C-7′), and 14.46 (C-8′). HR-MS for C_17_H_21_IO_3_ [M + H]^+^ *m/z*: Calcd 401.0608; Observed 401.0601. The major mass fragment observed was C_9_H_6_IO_3_^+^(288).

#### 3-Iodo-7-nonyloxychromone (4k)

It was obtained as white solid in 84% yield; m.p.: 78–80°C, R_f_: 0.56 (ethyl acetate: hexane, 3:7). IR (cm^−1^): 3,080 (aromatic C-H stretch), 2,918 (aliphatic C-H stretch), 1,636 (C=O stretch), 1,617 (aromatic C=C stretch), 1,587 (pyrone ring C=C stretch), 1,460 (C-H bending of CH_2_), and 1,264 (C-O Stretch). ^1^H NMR (400 MHz, CDCl_3_): *δ* 0.87 (3H, t, J = 6.8, H-9′), 1.26–1.82 (14H, m, H-2′, H-3′, H-4′, H-5′, H-6′, H-7′, and H-8′), 4.03 (2H, t, J = 6.4 Hz, H-1′), 6.79 (1H, d, J = 2 Hz, H-8), 6.95–6.97 (1H, m, H-6), 8.11 (1H, d, J = 8.8 Hz, H-5), and 8.18 (1H, s, H-2).^13^C NMR (100.6 MHz, CDCl_3_): 172.70 (C-4), 164.06 (C-7), 158.03 (C-9), 157.29 (C-2), 128.06 (C-5), 115.73 (C-6), 115.58 (C-10), 100.55 (C-8), 87.20 (C-3), 68.92 (C-1′), 31.94–22.73 (C-3′, C4′, C5′, C-6′, C-7′, C-8′), and 14.19 (C-9′). HR-MS for C_18_H_23_IO_3_ [M + H]^+^ *m/z*: Calcd 415.0764; Observed 415.0764. The major mass fragment observed was C_9_H_6_IO_3_^+^(288).

#### 7-Decyloxy-3-iodochromone (4l)

It was obtained as cream solid in 80% yield; m.p.: 68–71°C, R_f_: 0.46 (ethyl acetate: hexane, 3:7). IR (cm^−1^): 3,085 (aromatic C-H stretch), 2,925 (aliphatic C-H stretch), 1,641 (C=O stretch), 1,615 (aromatic C=C stretch), 1,591 (pyrone ring C=C stretch), 1,465 (C-H bending of CH_2_), and 1,278 (C-O Stretch). ^1^H NMR (400 MHz, CDCl_3_): *δ* 0.90 (3H, t, J = 6.8, H-10′), 1.30–1.82 (16H, m, H-2′, H-3′, H-4′, H-5′, H-6′, H-7′, H-8′ and H-9′), 4.02 (2H, t, J = 6.8 Hz, H-1′), 6.78 (1H, d, J = 2 Hz, H-8), 6.94–6.97 (1H, m, H-6), 8.10 (1H, d, J = 8.8 Hz, H-5), and 8.17 (1H, s, H-2). ^13^C NMR (100.6 MHz, CDCl_3_): 172.70 (C-4), 164.08 (C-7), 158.05 (C-9), 157.26 (C-2), 128.07 (C-5), 115.72 (C-6), 115.59 (C-10), 100.55 (C-8), 87.20 (C-3), 68.93 (C-1′), 31.96–22.75 (C-3′, C4′, C5′, C-6′, C-7′, C-8′, C-9′), and 14.19 (C-10′). HR-MS for C_19_H_25_IO_3_ [M + H]^+^ *m/z*: Calcd 429.0921; Observed 429.0946. The major mass fragment observed was C_9_H_6_IO_3_^+^(288).

#### 7-Dodecyloxy-3-iodochromone (4m)

It was obtained as white solid in 73% yield; m.p.: 58–60°C, R_f_: 0.46 (ethyl acetate: hexane, 3:7). IR (cm^−1^): 3,082 (aromatic C-H stretch), 2,922 (aliphatic C-H stretch), 1,639 (C=O stretch), 1,618 (aromatic C=C stretch), 1,589 (pyrone ring C=C stretch), 1,462 (C-H bending of CH_2_), and 1,268 (C-O Stretch). ^1^H NMR (400 MHz, CDCl_3_): *δ* 0.87 (3H, t, J = 6.8, H-12′), 1.26–1.83 (20H, m, H-2′, H-3′, H-4′, H-5′, H-6′, H-7′, H-8′, H-9′, H-10′ and H-11′), 4.02 (2H, t, J = 6.4 Hz, H-1′), 6.79 (1H, d, J = 2.4 Hz, H-8), 6.95–6.97 (1H, m, H-6), 8.11 (1H, d, J = 9.2 Hz, H-5), and 8.18 (1H, s, H-2). ^13^C NMR (100.6 MHz, CDCl_3_): 172.75 (C-4), 163.99 (C-7), 158.03 (C-9), 157.28 (C-2), 128.05 (C-5), 115.73 (C-6), 115.56 (C-10), 100 (C-8), 87.24 (C-3), 68.90 (C-1′), 31.94–22.74 (C-3′, C4′, C5′, C-6′, C-7′, C-8′, C-9′, C-10′, C-11′), and 14.20 (C-12′). HR-MS for C_21_H_29_IO_3_ [M + H]^+^ *m/z*: Calcd 457.1234; Observed 457.1205. The major mass fragment observed was C_9_H_6_IO_3_^+^(288).

#### 3-Iodo-7-tridecyloxychromone (4n)

It was obtained as white solid in 83% yield; m.p.: 67–70°C, R_f_: 0.46 (ethyl acetate: hexane, 3:7). IR (cm^−1^): 3,087 (aromatic C-H stretch), 2,920 (aliphatic C-H stretch), 1,643 (C=O stretch), 1,619 (aromatic C=C stretch), 1,599 (pyrone ring C=C stretch), 1,472 (C-H bending of CH_2_), and 1,273 (C-O Stretch). ^1^H NMR (400 MHz, CDCl_3_): *δ* 0.91 (3H, t, J = 5.6, H-13′), 1.28–1.87 (22H, m, H-2′, H-3′, H-4′, H-5′, H-6′, H-7′, H-8′, H-9′, H-10′, H-11′, and H-12′), 4.07 (2H, t, J = 5.2 Hz, H-1′),6.84 (1H, d, J = 2 Hz, H-8), 7.00–7.02 (1H, m, H-6), 8.16 (1H, d, J = 7.2 Hz, H-5), and 8.23 (1H, s, H-2). ^13^C NMR (100.6 MHz, CDCl_3_): 172.62 (C-4), 163.97 (C-7), 158.00 (C-9), 157.23 (C-2), 128.00 (C-5), 115.72 (C-6), 115.53 (C-10), 100.51 (C-8), 87.21 (C-3), 68.92 (C-1′), 32.00–22.78 (C-3′, C4′, C5′, C-6′, C-7′, C-8′, C-9′, C-10′, C-11′, C-12′), and 14.22 (C-13′). HR-MS for C_22_H_31_IO_3_ [M + H]^+^ *m/z:* Calcd 457.1234; Observed 457.1257. The major mass fragment observed was C_9_H_6_IO_3_^+^(288).

#### 3-Iodo-7-tetradecyloxychromone (4o)

It was obtained as white solid in 80% yield; m.p.: 67–70°C, R_f_: 0.43 (ethyl acetate: hexane, 3:7). IR (cm^−1^): 3,081 (aromatic C-H stretch), 2,928 (aliphatic C-H stretch), 1,647 (C=O stretch), 1,625 (aromatic C=C stretch), 1,588 (pyrone ring C=C stretch), 1,469 (C-H bending of CH_2_), and 1,277 (C-O Stretch). ^1^H NMR (400 MHz, CDCl_3_): *δ* 0.90 (3H, t, J = 5.6, H-14′), 1.25–1.84 (24H, m, H-2′, H-3′, H-4′, H-5′, H-6′, H-7′, H-8′, H-9′, H-10′, H-11′, H-12′, and H-13′), 4.05 (2H, t, J = 5.2 Hz, H-1′), 6.85 (1H, d, J = 2 Hz, H-8), 6.95–6.99 (1H, m, H-6), 8.18 (1H, d, J = 7.2 Hz, H-5), and 8.21 (1H, s, H-2). ^13^C NMR (100.6 MHz, CDCl_3_): 172.60 (C-4), 164.93 (C-7), 158.04 (C-9), 157.20 (C-2), 128.04 (C-5), 115.67 (C-6), 115.50 (C-10), 100.53 (C-8), 87.20 (C-3), 68.91 (C-1′), 32.01–22.77 (C-3′, C4′, C5′, C-6′, C-7′, C-8′, C-9′, C-10′, C-11′, C-12′, C-13′), and 14.18 (C-14′). HR-MS for C_23_H_33_IO_3_ [M + H]^+^ *m/z:* Calcd 485.1547; Observed 485.1533. The major mass fragment observed was C_9_H_6_IO_3_^+^(288).

#### 7-Bromo-3-iodochromone (4p)

It was obtained as dark brown solid in 79% yield; m.p.: 105–110°C, R_f_: 0.54 (ethyl acetate: hexane, 3:7). IR (cm^−1^): 3,073 (aromatic C-H stretch), 2,919 (aliphatic C-H stretch), 1,636 (C=O stretch), 1,614 (aromatic C=C stretch), 1,542 (pyrone ring C=C stretch), 1,438 (C-H bending of CH_2_), and 1,283 (C-O Stretch). ^1^H NMR (400 MHz, CDCl_3_): 7.41 (1H, d, J = 7.2 Hz, H-8), 7.80–7.82 (1H, m, H-7), 8.32 (1H, s, H-2), and 8.39 (1H, d, J = 2 Hz, H-5). ^13^C NMR (100.6 MHz, CDCl_3_): 172.07 (C-4), 158.11 (C-2), 155.44 (C-9), 137.74 (C-8), 132.53 (C-5), 124.43 (C-10), 120.18 (C-8), 118.17 (C-8), and 86.88 (C-3). HR-MS for C_9_H_4_BrIO_2_ [M + H]^+^ *m/z:* Calcd 350.8512; Observed 350.8529. The major mass fragment observed was C_9_H_5_O_2_^+^(145).

#### 6-Chloro-3-iodochromone (4q)

It was obtained as bright yellow solid in 85% yield; m.p.: 132–136°C, R_f_: 0.49 (ethyl acetate: hexane, 3:7). IR (cm^−1^): 3,076 (aromatic C-H stretch), 2,922 (aliphatic C-H stretch), 1,639 (C=O stretch), 1,618 (aromatic C=C stretch), 1,531 (pyrone ring C=C stretch), 1,444 (C-H bending of CH_2_), and 1,255 (C-O Stretch). ^1^H NMR (400 MHz, CDCl_3_): 6.93 (1H, d, J = 7.2 Hz, H-8), 7.15–7.27 (1H, m, H-7), 8.32 (1H, s, H-2), and 8.39 (1H, d, J = 2 Hz, H-5). ^13^C NMR (100.6 MHz, CDCl_3_): 172.37 (C-4), 159.73 (C-2), 154.51 (C-9), 134.75 (C-7), 130.64 (C-6), 124.63 (C-5), 122.25 (C-10), 121.21 (C-8), and 86.69 (C-3). HR-MS for C_9_H_4_ClIO_2_ [M + H]^+^ *m/z:* Calcd 306.9170; Observed 306.9163. The major mass fragment observed was C_9_H_5_O_2_^+^(145).

#### 6, 8-Dichloro-3-iodochromone (4r)

It was obtained as cream solid in 81% yield; m.p.: 143–145°C, R_f_: 0.61 (ethyl acetate: hexane, 3:7). IR (cm^−1^): 3,051 (aromatic C-H stretch), 2,923 (aliphatic C-H stretch), 1,641 (C=O stretch), 1,615 (aromatic C=C stretch), 1,538 (pyrone ring C=C stretch), 1,421(C-H bending of CH_2_), and 1,274 (C-O Stretch). ^1^H NMR (400 MHz, CDCl_3_): *δ* 7.78 (1H, d, J = 2 Hz, H-5), 8.14 (1H, d, J = 2 Hz, H-7), and 8.38 (1H, s, H-2). ^13^C NMR (100.6 MHz, CDCl_3_): 171.83 (C-4), 157.70 (C-2), 150.63 (C-9), 134.41 (C-7), 131.64 (C-6), 124.69 (C-5), 124.43 (C-10), 123.26 (C-8), and 86.91 (C-3). HR-MS for C_9_H_3_Cl_2_IO_2_ [M + H]^+^ *m/z:* Calcd 340.8627; Observed 340.8614. The major mass fragment observed was C_9_H_5_O_2_^+^(145).

#### 3-Iodo-6-methylchromone (4s)

It was obtained as yellow solid in 67% yield; m.p.: 110–116°C, R_f_: 0.55 (ethyl acetate: hexane, 3:7). IR (cm^−1^): 3,075 (aromatic C-H stretch), 2,921 (aliphatic C-H stretch), 1,642 (C=O stretch), 1,618 (aromatic C=C stretch), 1,540 (pyrone ring C=C stretch), 1,453 (C-H bending of CH_2_), and 1,267 (C-O Stretch). ^1^H NMR (400 MHz, CDCl_3_): 2.41 (3H, *CH*_*3*_), 6.28 (1H, d, J = 6 Hz, H-8), 7.32 (1H, d, J = 8.8 Hz, H-7), 7.81 (1H, d, J = 2 Hz, H-5), and 8.22 (1H, s, H-2). ^13^C NMR (100.6 MHz, CDCl_3_): 173.44 (C-4), 157.72 (C-2), 154.46 (C-9), 136.13 (C-6), 135.45 (C-7), 125.82 (C-5), 121.49 (C-10), 117.81 (C-8), 86.72 (C-3), and 21.09 (PhCH_3_). HR-MS for C_10_H_7_IO_2_ [M + H]^+^ *m/z:* Calcd 286.9563; Observed 286.9549. The major mass fragment observed was C_10_H_7_O_2_^+^(159).

#### 6-Fluoro-3-iodochromone (4t)

It was obtained as light yellow solid in 81% yield; m.p.: 123–126°C, R_f_: 0.57 (ethyl acetate: hexane, 3:7). IR (cm^−1^): 3,063 (aromatic C-H stretch), 2,942 (aliphatic C-H stretch), 1,647 (C=O stretch), 1,616 (aromatic C=C stretch), 1,553 (pyrone ring C=C stretch), 1,436 (C-H bending of CH_2_), and 1,257 (C-O Stretch). ^1^H NMR (400 MHz, CDCl_3_): 7.41 (1H, d, J = 7.2 Hz, H-8), 7.80–7.82 (1H, m, H-7), 8.32 (1H, s, H-2), and 8.39 (1H, d, J = 2 Hz, H-5). ^13^C NMR (100.6 MHz, CDCl_3_): 172.77 (C-4), 161.04 (C-6), 158.01 (C-2), 152.45 (C-9), 122.84 (C-7), 120.46 (C-8), 111.52 (C-5), and 86.69 (C-3). HR-MS for C_9_H_4_ClIO_2_ [M + H]^+^ *m/z:* Calcd 290.9262; Observed 290.9258. The major mass fragment observed was C_9_H_5_O_2_^+^(145).

#### 6-Chloro-3-iodo-7-methylchromone (4u)

It was obtained as light yellow solid in 83% yield; m.p.: 120–125°C, R_f_: 0.50 (ethyl acetate: hexane, 3:7). IR (cm^−1^): 3,067 (aromatic C-H stretch), 2,918 (aliphatic C-H stretch), 1,638 (C=O stretch), 1,612 (aromatic C=C stretch), 1,538 (pyrone ring C=C stretch), and 1,259 (C-O Stretch). ^1^H NMR (400 MHz, CDCl_3_): 2.47 (3H, *CH*_*3*_), 7.33 (1H, s, H-8), 8.15 (1H, s, H-5), and 8.23 (1H, s, H-2). ^13^C NMR (100.6 MHz, CDCl_3_): 172.37 (C-4), 159.73 (C-2), 154.51 (C-9), 145.53 (C-7), 131.33 (C-5), 129.12 (C-6), 123.01 (C-10), 120.13 (C-8), and 86.59 (C-3). HR-MS for C_10_H_6_ClIO_2_ [M + H]^+^ *m/z:* Calcd 320.9173; Observed 320.9158. The major mass fragment observed was C_10_H_7_O_2_^+^(159).

### Bioefficacy

#### Test Fungus

The test fungus *S. rolfsii* ITCC 6474 was procured from Indian Type Culture Collection (ITCC) center, Division of Plant Pathology, ICAR-Indian Agricultural Research Institute, New Delhi-110012, India and kept at 27°C for at least 4–7 days on Potato Dextrose Agar (PDA) slant. The fungus was subcultured in Petri plates for further bioassay studies.

#### *In vitro* Fungicidal Activity

A stock solution (1, 000 mgL^−1^) of each synthesized compound was prepared in DMSO. Preliminary screening was carried out at different concentrations. A final bioassay was conducted at five different concentrations namely 1,000, 500, 250, 125, and 62.50 mg L^−1^ of 4i–4n, 4p, 4s, and 4u chromones, and all other chromones were tested at 100, 50, 25, 12.5, and 6.25 mg L^−1^, respectively. All concentrations were tested in triplicates. Commercially available fungicide Mancozeb (technical) was taken as positive control.

An *in vitro* antifungal bioassay was carried out on PDA medium by poisoned food technique (Nene and Thapliyal, 1979). Fungal growth (colony diameter) was measured and percentage inhibition was calculated by Abbott’s formula (Abbott, 1925).

Percentage inhibition(I)=(C−T)×100/C,

where C = colony diameter (mm) of the control and T = colony diameter (mm) of the test plate.

Corrected percentage inhibition (IC) was calculated by given formula.

IC = (I−CF)/(100−CF)×100,

where I = Percentage inhibition, CF = (90-C)/C × 100, 90 is the diameter (mm) of the Petri plate, and C is the growth of the fungus (mm) in control.

ED_50_ (mg L^−1^) values (Effective Dose for 50% inhibition) were calculated using SPSS statistical Package (v16.0).

### Quantitative Structure Activity Relationship

QSAR analysis was done by taking negative logarithm of observed ED_50_ (mgL^−1^) [pED_50_ = −log (ED_50_)] as dependent variable and 2D descriptors (Table 1) as independent variables. 2D Structures of compounds were drawn in Chemdraw Ultra 7.0 software and converted to 3D structures. A total of 239 2D descriptors were determined encoding different molecular structural characteristics consisting of electronic, spatial, thermodynamic, and structural descriptors, for example, element count, atomic valence connectivity index (chiV), path cluster, estate number, retention index (chi), chain path count, logP, semi-empirical path count, molecular cluster, molecular weight, topological index, and refractivity. Descriptors were calculated by geometry optimization and energy minimization carried out by the batch energy minimization method in the Merck molecular force field (MMFF) at RMS gradient (criteria for convergence) 0.01, distance dependent dielectric 1, and the number of cycles (max) 1, 000. Different Baumann alignment-independent (AI) descriptors were also calculated. All computational work was carried out with the help of VLifeMDS QSAR plus 4.6 software using the Lenovo PC with Windows 8.1 operating system and the Intel (R) Celeron (R) processor.

**TABLE 1:** Molecular descriptors used in the QSAR study.

Descriptor	Description
T_2_Cl_6	This is the count of number of double bonded atoms (i.e. any double bonded atom, T_2) separated from chlorine atom by 6 bonds in a molecule
T_2_F_6	This is the count of number of double bonded atoms (i.e. any double bonded atom, T_2) separated from fluorine atom by 6 bonds in a molecule
T_T_F_3	Number of atoms which are separated from fluorine atom by three bonds
DeltaEpsilonC	A measure of contribution of electronegativity
ZcompDipole	This descriptor signifies the z component of the dipole moment (external coordinates)

#### Training and Test Set

The entire data of 21 compounds were divided into a training set (14 compounds) and a test set (4 compounds), and 3 compounds were taken for validation with the help of the sphere exclusion method (Hudson et al., 1996). Unicolumn statistics were used to check the accuracy of selection of training and test sets, as the maximum value of the training set was greater than that of the test set and the minimum value of the training set was less than that of the test set (Table 2).

**TABLE 2:** Unicolumn statistics of training and test sets for fungicidal activity.

Set	Average	Max	Min	Std. dev	Sum
Training	−1.7971	−0.9300	−2.7100	0.4514	−25.1600
Test	−1.9925	−1.6800	−2.5100	0.3648	−7.9700

#### Regression Analysis

Regression analysis was done with three model building methods, MLR, PCR, and PLS. Various 2D descriptors were taken as independent variables and pED_50_ values as dependent variables by taking cross-correlation limit as 0.5; five variables in the final equation and r^2^ as the term selection criteria, F-test “in” at 4 and “out” at 3.99, r^2^ and F-test value. Values were fixed at 0 for variance cutoff, 10 for random iterations, and auto scaling for scaling. Developed QSAR models were assessed with the help of statistical parameters such as *n* = total number of compounds used in regression, k = total number of descriptors in a model, r^2^ = regression coefficient, q^2^ = cross-validated correlation coefficient, F = F-test (Fisher test value) for statistical significance, pred_r^2^ = predictive squared correlation coefficients, pred_r^2^se = coefficient of correlation of predicted data set, and r^2^ se and q^2^ se = standard error (SE) of estimation.

#### Multiple Linear Regression Analysis

MLR defines linear relationship between a single response variable and a number of predictor variables. In the present work, pED_50_ fungicidal activity was response variable and 2D descriptors were predictor variables. In this method, regression coefficients values (r^2^) were calculated by the least squares curve fitting method. In regression analysis, conditional mean of the dependent variable (pED_50_) Y depends on (descriptors) X (Eq. 1).

Y=b1x1+ b2x2+ b3x31+c (1)

where Y = dependent variable, “x”s = independent variables, “b”s = regression coefficients for “x”s, and “c” = regression constant or intercept (Devillers, 1996; Croux and Joossens, 2005).

#### Principal Component Regression Method

In this method, the whole data were divided into principal components (PCs), smaller sets having major details of the large set. The main aim of PCR is to find out the values of a dependent variable with the help of selected PCs of independent variables. These PCs were not correlated, but had a basic linear relationship of original variables. The data were rotated into a new set of axes in such a way that first few axes showed greatest variability within the data. First PC (PC1) had maximum possible variation in the data, and each successive PC was taken perpendicular to preceding PCs and represent highest of the outstanding variance. The PC value is calculated by rotation of each point to a particular axis. A new group of axes for the data was chosen on the basis of a descending value data variance. Principal component analysis (PCA) also describes the fashion of similarity of the observations and the variables by exhibiting them as points in maps. PCR gives a mechanism for obtaining structure in datasets.

#### Partial Least Squares Regression Method

The partial least squares (PLS) test correlation between a set of dependent variables (Y) and a set of predictor variables (X). The main aim of PLS regression is helpful in describing the common structure by estimating the biological activity (dependent variables Y) from descriptors (X) (Huberty, 1994). PLS developed orthogonal components based on the relationship between predictors and respective outputs, while retaining highest variance of independent variables.

### Validation of The QSAR Model

**FIGURE 1:**
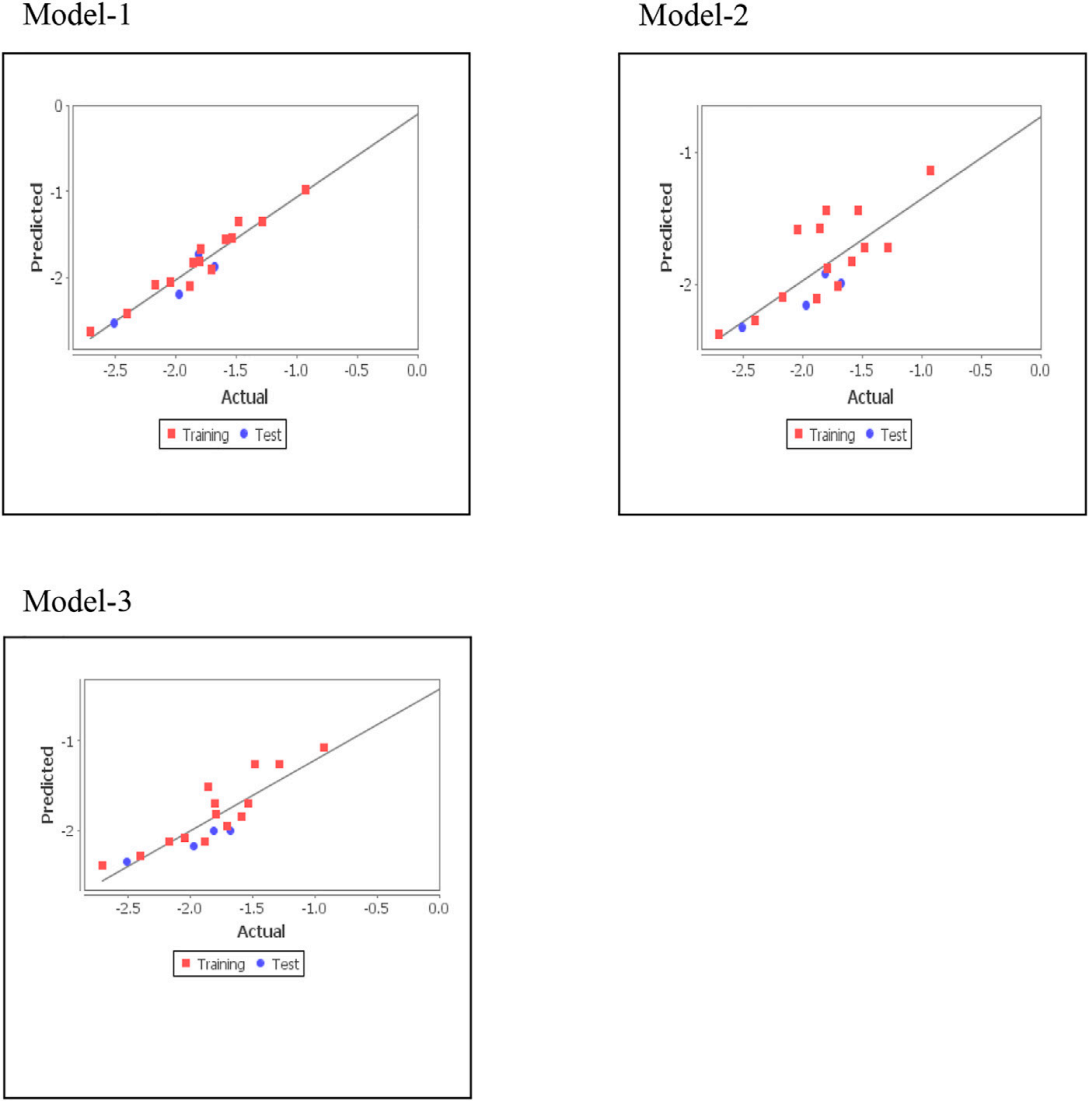
Graphs of experimental vs. predicted fungicidal activity of different models.

**FIGURE 2:**
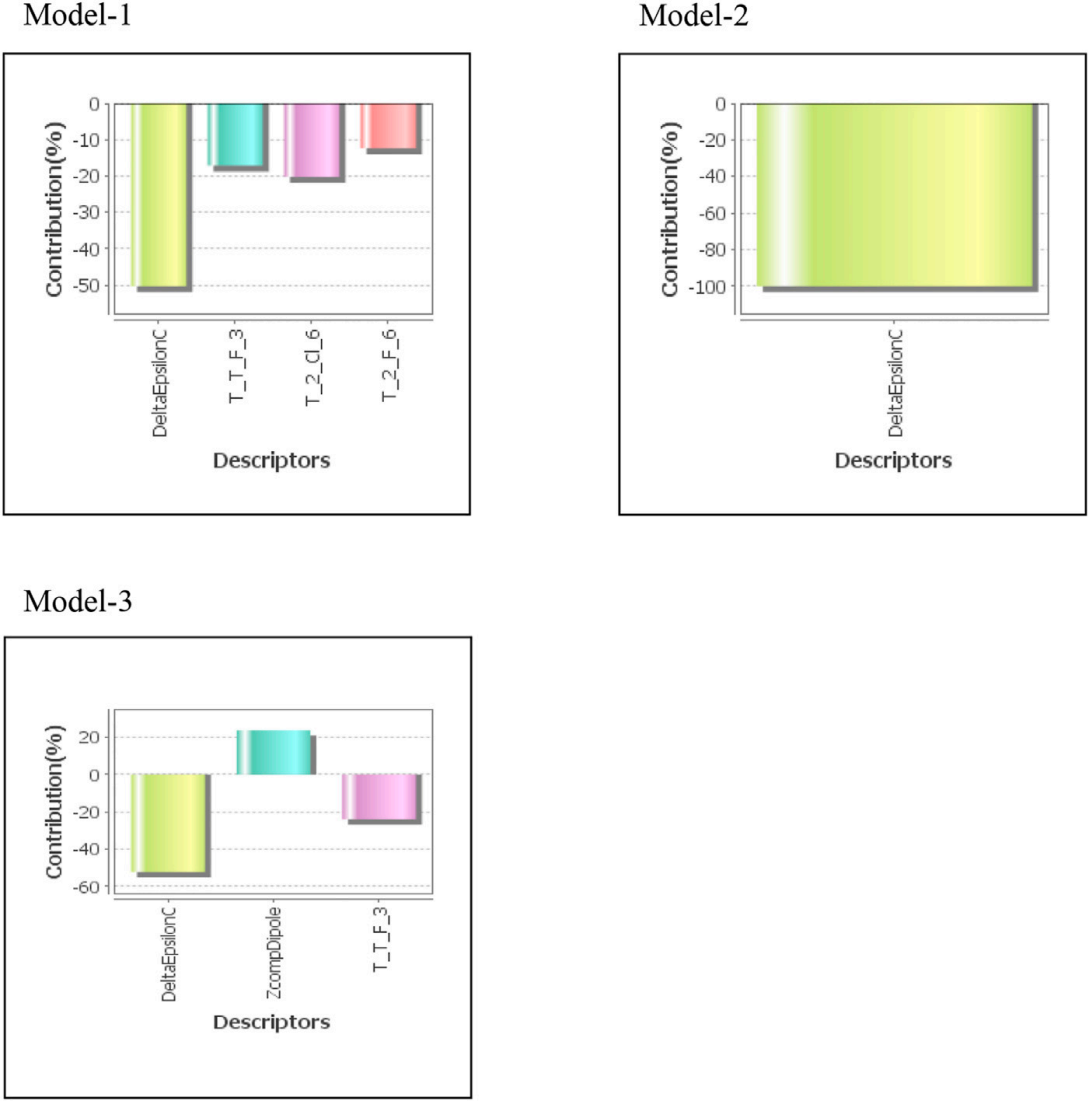
Contribution charts of the 2D-QSAR models.

The QSAR model was validated with Leave-one-out (LOO) cross validation, by dividing training dataset into equal size subsets after eliminating one biological activity data (number of subsets = number of data points). These subsets were used to develop the model for calculating predicted activity (value of response variable of excluded data). Since in LOO subset all the data points were serially considered as predicted, the mean value of predicted activity was similar for LOO q^2^ and r^2^. After elimination of the next data point, the same procedure was repeated until all data points were removed. Thus, three statistically significant models were developed by LOO cross-validation. (Kubyani, 1994). Eq. 2 was used for calculating q^2^.

$$q^{2}\mathrm{=1}-\sum {\left(Y_{\mathrm{pred}}-Y_{\mathrm{act}}\right)}^{2}/\sum \left(Y_{\mathrm{act}}-Y_{\mathrm{mean}}\right)$$ (2)

where Y_pred_ = predicted, Y_act_ = actual, Y_mean_ = mean values of the pED_50_, and Σ (Y_pred_ − Y_act_)^2^ = predictive residual error sum of squares (PRESS). External validation has also been performed to verify model validity, which tests how well the equation generalizes. A training set was used to develop an adjustment model for predicting activities of test set members. The predictive performance of equations was determined by q^2^, and coefficients of predictive squared correlation (pred _r^2^). pred_r^2^ was calculated by Eq. 3.

$$\mathrm{pred\_}r^{2}\mathrm{=1}-{\sum \left(Y_{\mathrm{pred}\left(\mathrm{Test}\right)}-Y_{\left(\mathrm{Test}\right)}\right)}^{2}\;/\sum \left(Y_{\mathrm{Test}}-Y_{\mathrm{Training}}\right)$$ (3)

where Y_pred(Test)_ = predicted activity and Y_Test_ = observed activity of test set compounds and Y_Training_ = mean activity value of the training set. Statistical significance of model was validated by the fitness plot (Figure 1) and it was also supported by closeness of observed and predicted activity (Table 3). The magnitude of different descriptors employed for developing QSAR models were present in contribution charts (Figure 2).

**TABLE 3:** Experimental and predicted fungicidal activity of chromones.

Test compound	ED_50_ (mgL^−1^)^a^	Fiducial limit		χ^2^	Experimental _P_ED_50_ ^b^	Predicted _P_ED_50_		
		Lower	Upper			MLR	PLS	PCR
4a	50.38	32.04	104.22	0.188	−1.70	−1.90	−1.95	−2.00
4b	19.12	14.68	24.20	4.42	−1.28	−1.35	−1.27	−1.72
4c	30.2	19.56	50.60	0.167	−1.48	−1.35	−1.27	−1.72
4d	39.28	25.84	72.61	0.178	−1.59	−1.55	−1.84	−1.83
4e	47.59	31.14	95.83	1.45	−1.68	−1.87	−2.00	−1.99
4f	61.23	23.88	302.70	5.41	−1.79	−1.66	−1.83	−1.88
4g	64.02	39.74	162.65	0.035	−1.81	−1.72	−2.01	−1.91
4h	75.58	47.05	190.60	0.223	−1.88	−2.09	−2.12	−2.11
4i	93.17	56.21	259.27	0.252	−1.97	−2.19	−2.17	−2.15
4j	124.7	71.91	391.47	0.314	−2.10	−2.27	−2.22	−2.20
4k	165.7	91.80	563.30	0.433	−2.22	−2.34	−2.25	−2.23
4l	248.6	139.68	742.55	1.724	−2.40	−2.41	−2.29	−2.27
4m	325	173.3	1,116.70	1.61	−2.51	−2.52	−2.35	−2.32
4n	410	200.45	1811.12	1.134	−2.61	−2.57	−2.37	−2.35
4o	515.4	234.53	2,856.32	0.445	−2.71	−2.61	−2.40	−2.37
4p	72.86	1.02	51.83	123.68	−1.86	−1.82	−1.52	−1.57
4q	63.75	49.76	89.08	2.88	−1.80	−1.80	−1.70	−1.44
4r	8.43	5.23	11.59	1.669	−0.93	−0.97	−1.07	−1.14
4s	147.8	101.10	272.12	4.87	−2.17	−2.07	−2.13	−2.09
4t	33.98	22.86	56.64	0.219	−1.53	−1.53	−1.70	−1.44
4u	110.9	40.19	133.05	0.124	−2.04	−2.04	−2.09	−1.59

^a^ The measured *in vitro* fungicidal activity against *s. rolfsii*

^b^ The negative logarithm of the measured ED_50_ (mg L^−1^).

Mancozeb ED_50_ = 17.17 mg L^−−1^.

## Result and Discussion

### Synthesis and Characterization

In this study, total 21 compounds (4a–4u) were synthesized out of which 10 compounds **(4f, 4g, 4h, 4i, 4j, 4k, 4l, 4m, 4n, and 4o)** were novel. The compounds synthesized by the above method were obtained in the yield ranging from 67 to 89%. Characterization of compounds was done by IR, ^1^H NMR ^13^C NMR, and LC-HRMS. In enaminones (3a–3u), peaks at *δ* 5.57–5.73 (1H, d, J = 8.8 Hz, H-2) and *δ* 7.55–7.92 (1H, d, J = 8.8 Hz, H-3) as two doublets for two protons with J = 8.8 Hz each were representative peaks of olefinic bond in ^1^H-NMR spectrum of all the compounds, and confirms the formation of enaminone. In ^13^C-NMR, the peaks at *δ* 89.75–89.93 (C-2) and 154.01–158.67 (C-3) for HC = CH and at *δ* 190.31–190.90 for C=O were conspicuous for all the compounds. The higher chemical shifts values of H-3 and C-3 than H-2 and C-2 were due to carbonyl moiety, which polarizes the C=C double bond. In IR spectra, stretching of (C=O) at 1,628–1,647 and (C=C) at 1,539–1,593 cm^−1^ supported the NMR data.

In case of chromones (4a–4u), peaks at *δ* 7.93–8.38 (1H, s, H-2) in ^1^H NMR and 157.20–163.56 (C-2) and 171.83–175.44 (C-4, C=O) in ^13^C NMR spectra of all compounds confirms synthesis of chromone derivatives. Stretching of (C=O) at 1,635–1,647 and (pyrone ring, C=C) at 1,531–1,599 cm^−1^ in IR spectra justified the NMR data.

### *In Vitro* Fungicidal Activity

**FIGURE 3:**
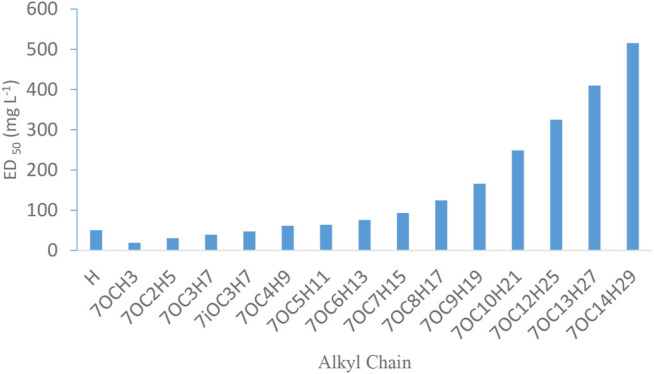
Effect of increase in alkyl chain on fungicidal activity of chromones.

*In vitro* evaluation showed that all the tested compounds (4a–4u) exhibited promising fungicidal activity against *S. rolfsii* (Table 3), and compound **4r** was found to be most active (ED_50_ = 8.43 mgL^−1^) which was better than Mancozeb (ED_50_ = 17.17 mgL^−1^), a commercial fungicide. It was observed that with increasing length of alkyl chain, fungicidal activity of alkoxy chromones (4a–4n) significantly decreased (Figure 3). Among halo chromone derivatives **(4p, 4q, 4r, 4t, and 4u)** 6, 8 dichlro derivative **(4r)** exhibited the highest and 4 fluoro derivative **(4u)** the least activity.

### 2D-QSAR Study

Three statistically significant QSAR models *viz*. Model-1 (MLR) Eq. 4, Model-2 (PCR) Eq. 5, and Model-3 (PLS) Eq. 6 were developed in 2D-QSAR study of fungicidal activity.

#### Model-1 (MLR)

$$\mathrm{pE}d_{\mathrm{50}}=-\mathrm{25}\mathrm{.5825}\left(\mathrm{DeltaEpsilonC}\right)-0\mathrm{.2256}\left(\mathrm{T\_T\_F\_3}\right)-0\mathrm{.7476}\left(\mathrm{T\_2\_Cl\_6}\right)-0\mathrm{.2716}\left(\mathrm{T\_2\_F\_6}\right)-3\mathrm{.5771}$$ (4)

where *n* = 14, DF = 9, r^2^ = 0.9434, q^2^ = 0.9117, F_test = 37.5361, r^2^_se = 0.1290, q^2^_se = 0.1612, pred_r^2^ = 0.8374, and pred_r^2^se = 0.1730.

#### Model-2 (PCR)

$$pEd_{50}=-\;13.2406\;\left(\text{DeltaEpsilonC}\right)-2.8722$$ (5)

where *n* = 14, DF = 12, r^2^ = 0.5979, q^2^ = 0.4509, F_test = 17.8453, r^2^_se = 0.2979, q^2^_se = 0.3482, pred_r^2^ = 0.6877, and pred_r^2^se = 0.2397.

#### Model-3 (PLS)

$$pEd_{50}=13.5036\;\left(\text{DeltaEpsilonC}\right)+130.1390\;\left(\text{ZcompDipole}\right)-\;0.1607\;\left(\text{T}\_\text{T}\_\text{F}\_3\right)-2.8393$$ (6)

where *n* = 14, DF = 11, r^2^ = 0.8006, q^2^ = 0.6167, F_test = 22.0866, r^2^_se = 0.2191, q^2^_se = 0.3038, pred_r^2^ = 0.6186, and pred_r^2^se = 0.2649.

In above QSAR models, correlation coefficient (r^2^) was used to calculate biological activity variance by multiplying with 100. The predictive ability (q^2^) of generated QSAR models was assessed by LOO (Left-out-one) method. F is the ratio of variance of models and that of error in regression. Models with a higher F value and lower SE of estimation (r^2^se and q^2^se) were considered statistically significant. External validation with pred_r^2^ > 0.3, established the predictive power of the QSAR model. Among these three models, the MLR model was found best as revealed by q^2^, r^2^, higher values of F-test, and pred_r^2^. The high q^2^ value is the best indicator of 2D QSAR’s reliability since only a high r^2^ could be due to data overfitting. Quite often, a q^2^ value of more than 0.5 is considered appropriate. (Golbraikh and Tropsha, 2002; Doweyko, 2004; Ponce et al., 2004).

The developed models showed that fungicidal activity was inversely related to descriptors, DeltaEpsilonC and AI descriptor, T_2_Cl_6, T_2_F_6, and T_T_F_3 and directly related to ZcompDipole. Two descriptors *viz.* DeltaEpsilonC and T_2_Cl_6 significantly (∼70%) impact the fungicidal activity of test compounds. Alignment Independent (AI) descriptors were estimated, as explained in Baumann’s paper (Balaban, 1982), on the basis of molecular topology, type of bond, and atom. Every atom was given a minimum of one and a maximum of three attributes. Molecular topology (T) was designated as the first attribute, followed by atom symbol and atoms linked with multiple (double or triple) bonds as second and third attribute, respectively. Then, selective distance count statistics, which counts all the fragments between the first atom and the last atom isolated by a graph distance, for all combinations of various attributes were calculated. Graph distance is the least number of atoms across the path joining two atoms in molecular structure. For example, selective distance count statistic “AB2” (e.g., TOPO2N3) counts all the fragments between a start atom with attribute “A” (e.g., “2” a double bonded atom) and an end atom with attribute “B” (e.g., “N”) separated by a graph distance 3. Topological indices are numerical values associated with chemical constitutions which establish correlation between biological activity and chemical structure. AI descriptors in this study were calculated with the help of attributes namely, 2 (atom with double bond), 3 (atom with double bond), C (Carbon), N (Nitrogen), O (Oxygen), S (Sulfur), H (Hydrogen), F (Fluorine), Cl (Chlorine), and Br (Bromine) with distance ranging from 0 to 7. DeltaEpsilonC is a measure of contribution of electronegativity. The result revealed that it is negatively correlated with fungicidal activity of the test compounds.

## Conclusion

The study revealed that all test compounds showed fungicidal activity against *S. rolfsii*., but compound **4r** showed the highest activity. The QSAR study determined quantitative correlation between fungicidal activity and structural/physicochemical properties of test compounds. The variables in developed model equations established that structural, molecular shape analysis, electronic, and thermodynamic descriptors played a major role in fungicidal activity of the compounds. In the case of MLR and PLS, the overall prediction was found to be around 94 and 80%, respectively. The 2D-QSAR study revealed that results of MLR analysis exhibited significant predictive power and reliability than the other two methods (PCR and PLS). Information and understanding of descriptors influencing fungicidal activity of these chromones could be used for structure optimization to improve activity.

## Data Availability

The original contributions presented in the study are included in the article/Supplementary Material; further inquiries can be directed to the corresponding author.
