# Supplementary material for: Synthesis, Biological Evaluation, and QSAR Studies of 3-Iodochromone Derivatives as Potential Fungicides
Source: Front Chem. 2021 Apr 30;9:636882. doi: 10.3389/fchem.2021.636882 (PMC8120269; doi:10.3389/fchem.2021.636882)
Supplement: Supplementary file 1 [file DataSheet1.docx]

**Synthesis, biological evaluation and QSAR studies of 3-Iodochromone derivatives as potential fungicides**

Parshant Kaushik^1, *^, Najam A. Shakil^1^ and Virendra S. Rana^1^

*^1^Division of Agricultural Chemicals, ICAR-Indian Agricultural Research Institute, New Delhi, India*

*Correspondence:parshantagrico@gmail.com, parshant.kaushik@icar.gov.in

**Supporting Information**

**Figure 1:** ^1^H NMR spectrum of compound **4b**

**Figure 2:** ^13^C NMR spectrum of compound **4b**

**Figure 3:** ^1^H NMR spectrum of compound **4n**

**Figure 4:** ^13^C NMR spectrum of compound **4n**

**Figure 5:** ^1^H NMR spectrum of compound **4r**

**Figure 6:** ^13^C NMR spectrum of compound **4r**


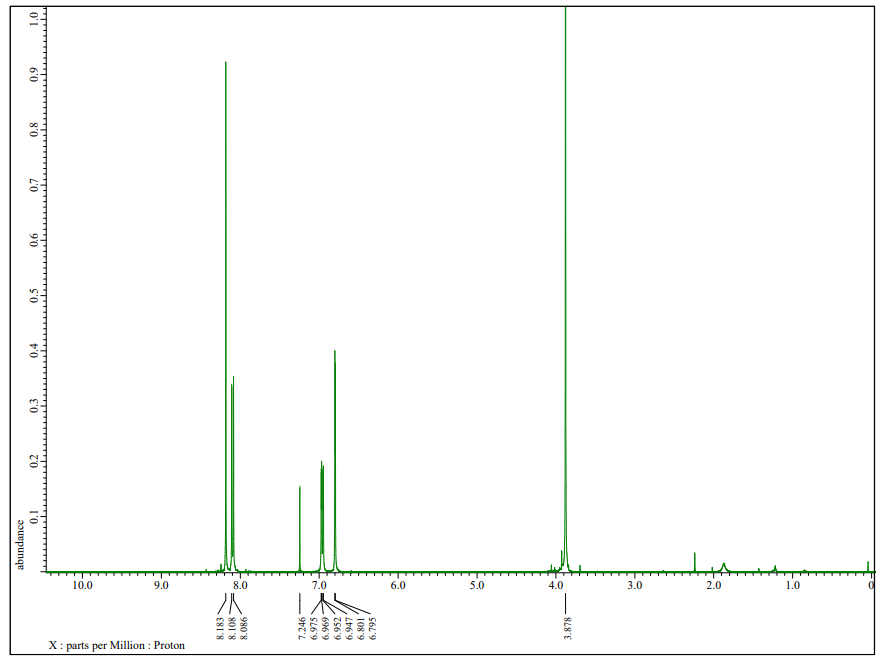


**Figure 1:** ^1^H NMR (400 MHz, CDCl_3_) of compound **4b**


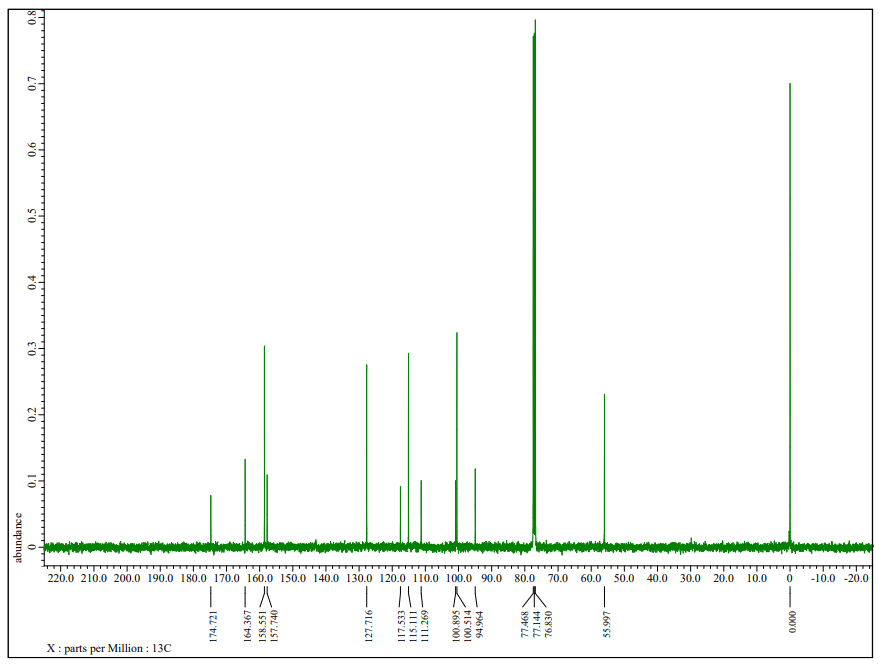


**Figure 2**: ^13^C NMR (100.6 MHz, CDCl_3_) of compound **4b**


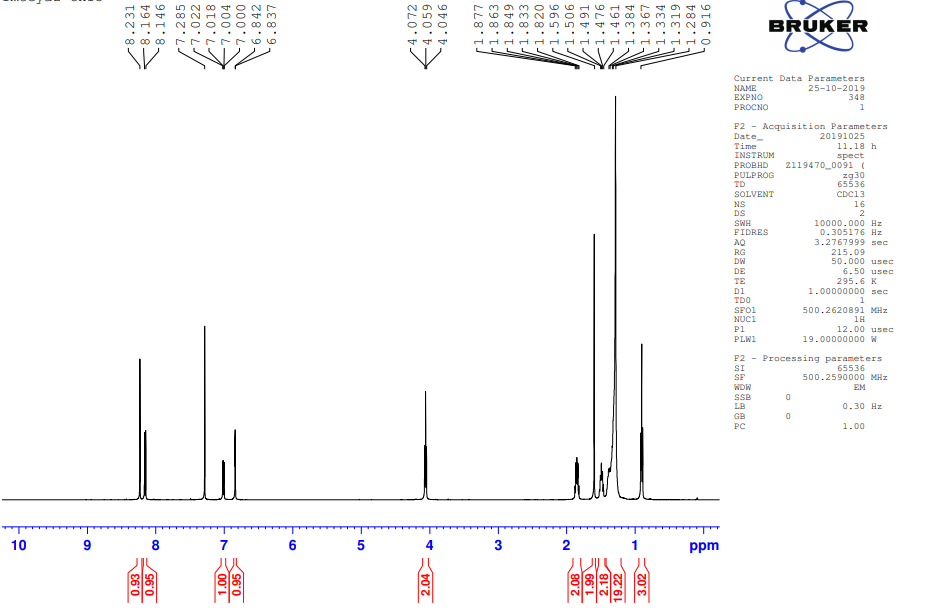


**Figure 3**: ^1^H NMR (400 MHz, CDCl_3_) of compound **4n**


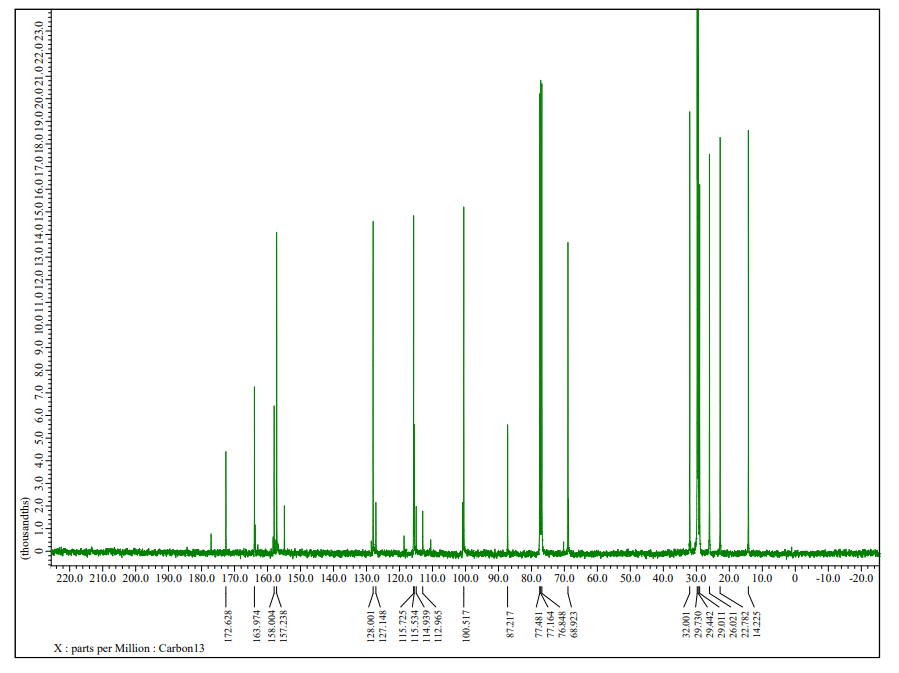


**Figure 4**: ^13^C NMR (100.6 MHz, CDCl_3_) of compound **4n**


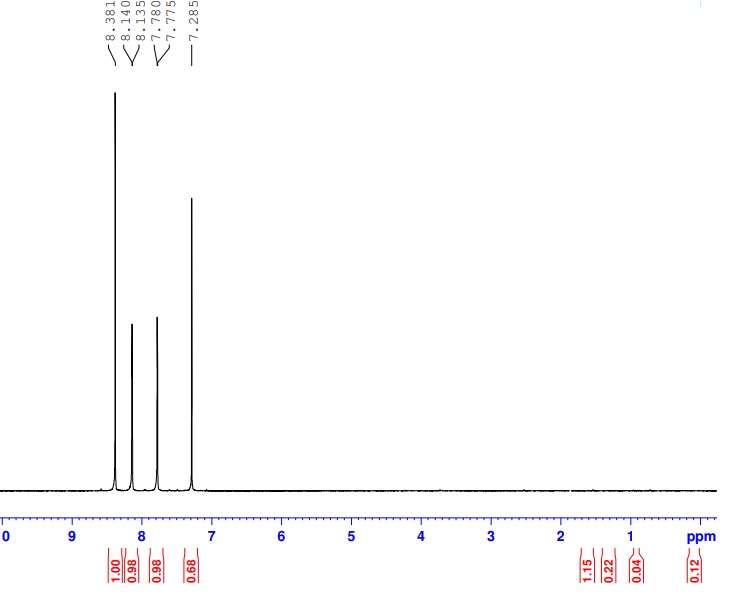


**Figure 5**: ^1^H NMR (400 MHz, CDCl_3_) of compound **4r**


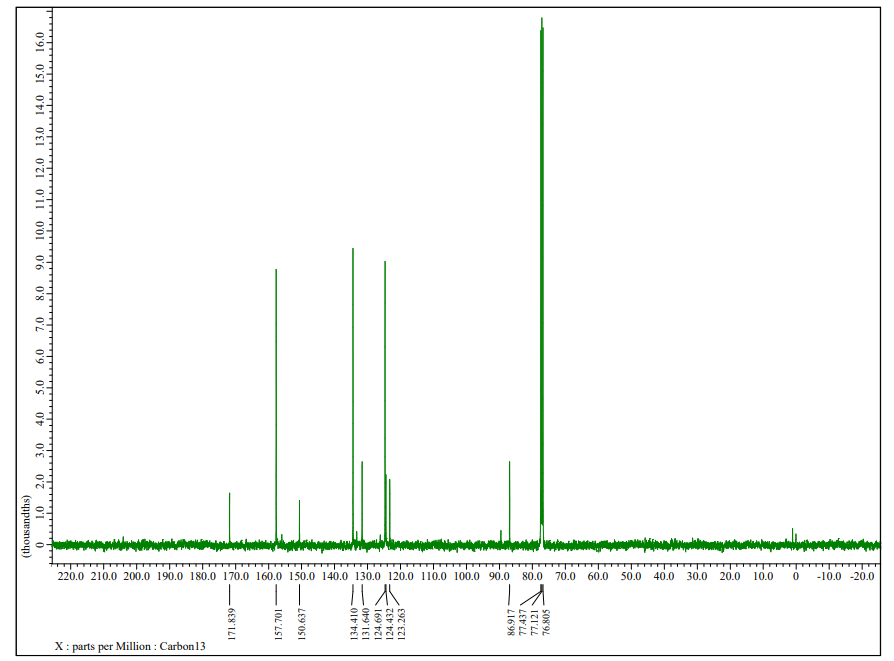


**Figure 4**: ^13^C NMR (100.6 MHz, CDCl_3_) of compound **4r**
